# Dual inhibition of DNA-PK and DNA polymerase theta overcomes radiation resistance induced by p53 deficiency

**DOI:** 10.1093/narcan/zcaa038

**Published:** 2020-12-21

**Authors:** Rashmi J Kumar, Hui Xiao Chao, Dennis A Simpson, Wanjuan Feng, Min-Guk Cho, Victoria R Roberts, Aurora R Sullivan, Sonam J Shah, Anne-Sophie Wozny, Katerina Fagan-Solis, Sunil Kumar, Adam Luthman, Dale A Ramsden, Jeremy E Purvis, Gaorav P Gupta

**Affiliations:** Lineberger Comprehensive Cancer Center, University of North Carolina at Chapel Hill, Chapel Hill, NC 27599, USA; Curriculum in Genetics and Molecular Biology, University of North Carolina at Chapel Hill, Chapel Hill, NC 27599, USA; Curriculum in Bioinformatics and Computational Biology, University of North Carolina at Chapel Hill, Chapel Hill, NC 27599, USA; Lineberger Comprehensive Cancer Center, University of North Carolina at Chapel Hill, Chapel Hill, NC 27599, USA; Lineberger Comprehensive Cancer Center, University of North Carolina at Chapel Hill, Chapel Hill, NC 27599, USA; Lineberger Comprehensive Cancer Center, University of North Carolina at Chapel Hill, Chapel Hill, NC 27599, USA; Lineberger Comprehensive Cancer Center, University of North Carolina at Chapel Hill, Chapel Hill, NC 27599, USA; Lineberger Comprehensive Cancer Center, University of North Carolina at Chapel Hill, Chapel Hill, NC 27599, USA; Lineberger Comprehensive Cancer Center, University of North Carolina at Chapel Hill, Chapel Hill, NC 27599, USA; Lineberger Comprehensive Cancer Center, University of North Carolina at Chapel Hill, Chapel Hill, NC 27599, USA; Lineberger Comprehensive Cancer Center, University of North Carolina at Chapel Hill, Chapel Hill, NC 27599, USA; Lineberger Comprehensive Cancer Center, University of North Carolina at Chapel Hill, Chapel Hill, NC 27599, USA; Lineberger Comprehensive Cancer Center, University of North Carolina at Chapel Hill, Chapel Hill, NC 27599, USA; Department of Biochemistry and Biophysics, University of North Carolina at Chapel Hill, Chapel Hill, NC 27599, USA; Lineberger Comprehensive Cancer Center, University of North Carolina at Chapel Hill, Chapel Hill, NC 27599, USA; Department of Biochemistry and Biophysics, University of North Carolina at Chapel Hill, Chapel Hill, NC 27599, USA; Curriculum in Bioinformatics and Computational Biology, University of North Carolina at Chapel Hill, Chapel Hill, NC 27599, USA; Computational Medicine Program, University of North Carolina at Chapel Hill, Chapel Hill, NC 27599, USA; Department of Genetics, University of North Carolina at Chapel Hill, Chapel Hill, NC 27599, USA; Lineberger Comprehensive Cancer Center, University of North Carolina at Chapel Hill, Chapel Hill, NC 27599, USA; Curriculum in Genetics and Molecular Biology, University of North Carolina at Chapel Hill, Chapel Hill, NC 27599, USA; Department of Biochemistry and Biophysics, University of North Carolina at Chapel Hill, Chapel Hill, NC 27599, USA; Department of Radiation Oncology, University of North Carolina at Chapel Hill, Chapel Hill, NC 27599, USA

## Abstract

*TP53* deficiency in cancer is associated with poor patient outcomes and resistance to DNA damaging therapies. However, the mechanisms underlying treatment resistance in p53-deficient cells remain poorly characterized. Using live cell imaging of DNA double-strand breaks (DSBs) and cell cycle state transitions, we show that p53-deficient cells exhibit accelerated repair of radiomimetic-induced DSBs arising in S phase. Low-dose DNA-dependent protein kinase (DNA-PK) inhibition increases the S-phase DSB burden in p53-deficient cells, resulting in elevated rates of mitotic catastrophe. However, a subset of p53-deficient cells exhibits intrinsic resistance to radiomimetic-induced DSBs despite DNA-PK inhibition. We show that p53-deficient cells under DNA-PK inhibition utilize DNA polymerase theta (Pol θ)-mediated end joining repair to promote their viability in response to therapy-induced DSBs. Pol θ inhibition selectively increases S-phase DSB burden after radiomimetic therapy and promotes prolonged G2 arrest. Dual inhibition of DNA-PK and Pol θ restores radiation sensitivity in p53-deficient cells as well as in p53-mutant breast cancer cell lines. Thus, combination targeting of DNA-PK- and Pol θ-dependent end joining repair represents a promising strategy for overcoming resistance to DNA damaging therapies in p53-deficient cancers.

## INTRODUCTION

*TP53* (gene product p53) is the most commonly mutated tumor suppressor gene (1). p53 mediates pleiotropic tumor-suppressive effects through regulation of cell cycle arrest, apoptosis and cellular metabolism in response to cellular stress (2,3). Beyond its role as a tumor suppressor, p53 deficiency is associated with poor prognostic outcomes across many different cancer types (4–6). Furthermore, there is accumulating clinical and preclinical evidence that p53 deficiency in cancer is often associated with resistance to a variety of DNA damaging therapies (4,7–11). Nonetheless, the mechanisms underlying therapeutic resistance in p53-deficient cells remain poorly characterized. Several factors play into the ambiguity surrounding the role of p53 and radioresistance. Early work suggested a role for loss of p53-mediated apoptosis in enabling increased survival post-radiation (9,12). However, in epithelial cancer cell models, p53-induced cell cycle arrest, rather than apoptosis, has been associated with radiosensitization (13). Yet, p53-mediated effects distinct from cell cycle arrest and apoptosis may also regulate radiosensitivity, as critical aspects of this relationship seem to be independent of p21 induction and the G1/S checkpoint (14–16).

Modulation of DNA double-strand break (DSB) repair by p53 may also determine radiosensitivity. Despite extensive study, the impact of p53 status on DSB repair and implications for radiosensitization remain controversial and likely context dependent (17,18). Colocalization of p53 to sites of DNA damage suggests that both direct and indirect modulation of repair are plausible (19). Several studies have demonstrated a role for p53 in suppressing homologous recombination (HR) repair, possibly through direct interactions with RPA and/or Rad51 (20,21). Consistent with these observations, p53-deficient HCT116 cells exhibit hyperactive HR activity and resistance to topoisomerase inhibitor therapy (22). p53 also regulates nonhomologous end joining (NHEJ), although the observed effects are highly dependent on the type of DSBs induced and the assays used to measure repair. Wild-type p53 seems to promote error-free repair by NHEJ, possibly through re-annealing of complementary single-stranded DNA (ssDNA) overhangs at the DSB (23,24). In contrast, expression of mutant p53 accelerates global DSB end joining rates and also promotes error-prone microhomology-mediated end joining (MMEJ) (25–27). How these regulatory effects of p53 on DSB repair modulate radiation resistance remains poorly resolved. However, as inhibitors of DNA-dependent protein kinase (DNA-PK) and DNA polymerase theta (Pol θ, the predominant mediator of MMEJ in mammals) are in clinical investigation and/or development, an improved understanding of end joining repair pathways in radioresistance may inform optimized therapeutic strategies (28–30).

In this study, we investigate the relationship between radiomimetic-induced DNA damage and cell fate at the single-cell level upon induced p53 deficiency in an epithelial cell model using time-lapse microscopy of cell cycle and DNA damage biosensors. We find that p53-deficient cells exhibit accelerated resolution of DNA damage foci, particularly in S phase of the cell cycle. We show that the accelerated resolution of radiomimetic-induced DNA damage in p53-deficient cells is dependent on DNA-PK, a critical serine/threonine kinase in the NHEJ pathway (31). Inhibition of DNA-PK partially restores sensitivity to DSB-inducing agents in p53-deficient cells, with therapy-resistant cells exhibiting residual DSB repair activity. We further identify Pol θ-mediated end joining (TMEJ) as a salvage DSB repair pathway that confers replicative viability and therapeutic resistance in p53-deficient cells. Thus, our work recognizes a critical role for two targetable end joining repair pathways—NHEJ and TMEJ—in mediating resistance to DNA damaging therapy in p53-deficient cells.

## MATERIALS AND METHODS

### Key reagents

All key reagents can additionally be found with catalog number and identifiers in Supplementary Table S1, as well as detailed information on software used for analyses and algorithms available for image processing.

### Cell culture

*T* *P53*^+/+^, *Fusion-Reporter* (*TP53*^+/^^+^, *PCNA-mCherry*, *53BP1-mVenus*), *TP53^−^^/^^−^* and *TP53^−^^/^^−^POLQ^−/−^* cells are hTERT immortalized RPE1 (ATCC). The fusion-reporter cell line was gifted by Dr Jeremy Purvis and originally created utilizing lentiviral transduction of the dual reporters into RPE1 followed by single-clone selection for stably expressing cells. Cells were maintained in Dulbecco’s modified Eagle’s medium (DMEM), with 10% fetal bovine serum (Hyclone FBS) and 2 mM l-glutamine (Thermo Fisher). All cells were maintained at 37°C in an atmosphere of 5% CO_2_. Cells were routinely tested for mycoplasma contamination using PlasmoTest (Invivogen).

### Establishment of stable cell lines

To create the *TP53*- and *POLQ*-deficient cell lines, we used the Alt-R–CRISPR–Cas9 system (IDT). We performed neon transfection (Invitrogen) and followed the manufacturer’s protocol with Alt-R HiFi Cas9 nuclease, crRNA and tracrRNA purchased from IDT. crRNA was designed using MIT CRISPR (http://crispr.mit.edu) to target exon 2 of the *TP53* gene to create the *TP53*^−/−^ cell line and the polymerase domain of the *POLQ* gene to create the *TP53*^−^^/^^−^*POLQ*^−^*^/−^* cell line. Forty-eight hours after transfection, cells were seeded for single-clone selection. Restriction enzyme screening, western blots, PCR screening and Sanger sequencing confirmed gene targeting, as well as functional assays.

### Immunofluorescence

Cells were fixed with 3% paraformaldehyde (PFA) for 15 min at room temperature, followed by permeabilization with 0.25% Triton X-100 in phosphate-buffered saline (PBS). Blocking with 1% bovine serum albumin (BSA) in PBS was used prior to immunostaining experiments using the antibodies listed below. Nuclei were visualized by staining with DAPI. The primary antibodies used were γH2AX (1:500, Trevigen, 4418-APC-100) and 53BP1 (1:500 for immunofluorescence, Bethyl, #A300-272A). The secondary antibodies were FITC Goat Anti-Mouse IgG (H + L) (1:500, Jackson ImmunoResearch, 115-095-003) and FITC Goat Anti-Rabbit IgG (H + L) (1:500, Jackson ImmunoResearch, 111-095-144). Images were acquired using the GE IN CELL 2200 high-throughput imaging system, or the Olympus BX61 fluorescence microscope at 40× magnification.

### siRNA treatment

WT Fusion-Reporter RPE1 cells were passaged twice after −80°C thaw and plated on 12-well plates at a density of 100,000 cells per well for siRNA treatment. Twenty-four hours after plating, cells were exposed to 10 nM per well si-TP53 (SMART pool from Dharmacon), and si-Control (non-targeting SMART pool from Dharmacon), in OPTIMEM with RNA-iMAX (Thermo Fisher) as a transfection reagent. As a no-treatment control, cells were exposed to RNA-iMAX and OPTIMEM without siRNA. Forty-eight hours after transfection, cells were transferred onto 12-well Cell-Tak-coated glass plates (Cellvis), at a concentration of 50,000 cells per well for imaging. Prior to imaging and at the end of imaging, samples were taken for RT-qPCR analysis of p53 mRNA to confirm siRNA knockdown. For the colony forming assay, MDA-MB-231 or BT-549 cells were plated on six-well plates at a density of 100,000 cells per well for siRNA treatment. Twenty-four hours after plating, cells were transfected twice for 2 days with 10 nM si-hPOLQ (SMART pool from Dharmacon) or si-Control. All the experiments were performed at least 48 h after siRNA transfection. See Supplementary Table S1 for details of siRNA information.

### Mixed competition assay—flow cytometry

*WT* Fusion-Reporter RPE1 cells and unlabeled *TP53^+/+^* (parental) or *TP53^−^^/^^−^* hTERT-RPE1 cell lines were plated on 96-well plates at a 1:1 ratio (1500 cells each for a total of 3000 cells per well), and irradiated after plating at 0, 2, 4 or 6 Gy, and left to grow. Irradiation was performed after cell plating to ensure time for cell adherence for both RPE1 and TP53^−/−^ RPE1. At indicated time points, cells were harvested by trypsinizing and quenching with PBS with 5% BSA. Cells were fixed with 2% PFA and subsequently transferred to V-bottom plates (Thermo Fisher, #249570). Cells were quantified by flow cytometry using the Intellicyt iQue at a volume of 100 μl per sample, collecting all events per well. For each condition, six biological replicates were collected.

### Time-lapse imaging microscopy

Cells stably expressing proliferating cell nuclear antigen (PCNA)-mCherry and tumor suppressor p53 binding protein 1 (53BP1)-mVenus were treated with siRNA for 48 h prior to imaging. PCNA-mCherry and 53BP1-mVenus fusion reporter is a gift from Dr. Jeremy Purvis and Hui Xiao Chao. Cells were plated on Cell-Tak (Corning)-coated glass-bottom 12-well plates (Cellvis) with phenol-free DMEM (Invitrogen) supplemented with 10% FBS and l-glutamine. Twenty-four hours after plating, cells were image captured every 10 min for 72 h in the mCherry and mVenus fluorescence channels. Eighteen hours into imaging, DNA-PK inhibitor (DNA-PKi, NU7441) was added at a concentration of 0.5 μM per well and/or neocarzinostatin (NCS, Sigma-Aldrich) at a concentration of 100 ng/ml. We commenced imaging every 10 min in both channels for another 48 h. Fluorescence images were obtained using a Nikon Ti Eclipse inverted microscope with a 40× objective and Nikon Perfect Focus (PFS) system to maintain focus during acquisition period. Cells were maintained at constant temperature (37°C) and atmosphere (5% CO_2_). Nikon’s NIS Elements AR software was utilized for image acquisition. Image analysis was performed on ImageJ–Fiji and Cell Profiler.

### Colony forming assays

Cell lines used in the assay are indicated in the figures. RPE1 cells were treated with NCS and/or DNA-PKi for 24 h, after which we performed a medium change. For all colony forming assays, cells were incubated for 10–14 days at 37°C to allow colony formation, as determined by the size of colonies in the untreated condition. Colonies were stained by Coomassie blue and/or crystal violet solution. MDA-MB-231 or BT-549 cells were transfected with si-hPOLQ versus si-Control (see the ‘siRNA treatment’ section) and 300–2000 cells per 100-mm dish in triplicate were reseeded in the growth medium 48 h after second siRNA treatment. Samples were taken for RT-qPCR analysis of POLQ mRNA to confirm siRNA knockdown. After 6 h, cells were irradiated at 0, 1, 2 or 4 Gy with or without 0.5 μM NU7441.

### RNA extraction and RT-qPCR

RNA was extracted using the RNeasy Plus Mini Kit (QIAGEN) following manufacturer’s instructions. For quantitative RT-PCR, RNA concentrations were determined with a spectrophotometer (BioDrop; Biochrom). RNA was reverse transcribed using Maxima First Strand cDNA Synthesis Kit (Maxima, Thermo Fisher). Two reverse transcription reactions were performed for each sample using 100 ng RNA. RT-qPCR assays were performed using Fast SYBR™ Green Master Mix (Thermo Fisher, #4385612) and run on QuantStudio 6 and 7 Flex Real-Time PCR Systems (Thermo Fisher). Cycling conditions were 95°C for 15 min, followed by 40 (two-step) cycles (95°C, 15 s; 60°C, 60 s). Primers are listed in Supplementary Table S1.

### Chromosomal DNA repair assay

Cell lines used in the assay are indicated in the figures. 5  ×  10^5^ cells were transfected with sgLBR2 and TracrRNA complexed Cas9 protein at final concentrations of 22 pmol (sgRNA:tracrRNA duplex) and 18 pmol (Cas9) per reaction, with neon transfection kit (Invitrogen) using two 1350 V, 30 ms pulses in a 10-μl chamber. Sixty hours after transfection, cells were harvested for genomic DNA extraction (Nucleospin, Takara Bio). Part of the gDNA was utilized for Sanger sequencing and TIDE analysis after amplification of the genomic LBR2 locus. For analysis of INDELs, 100 ng of gDNA was amplified using phased primers. These libraries were indexed with the Illumina dual combinatorial indices. Following pooling, 2 × 150 cycle sequencing was done on an Illumina iSeq100™. INDELs were identified by comparing the target reference sequence to the resulting sequence reads in the FASTQ files via a 10-nucleotide sliding window using the ScarMapper program.

### Extrachromosomal TMEJ assay

For extrachromosomal substrate experiments, we employed a 612-bp linear substrate with ends possessing 70-nt 3′ ssDNA overhangs and a 20-nt 5′ overhang containing a 5′ biotin (Integrated DNA Technologies). Substrate was incubated with a 25-fold molar excess of streptavidin prior to transfection. The sequence of the double-stranded portion, oligonucleotides for generating overhang-containing end structures, electroporation conditions and analysis methods are described in (29).

### Digital PCR

Primers and 5′ hydrolysis probes were designed to specifically detect the copies of lamin B receptor (*LBR*) locus. *ESR1* locus was used as genomic control. Each reaction assay contained 10 μl of 2× dPCR Supermix for Probes (no dUTP), 0.9 μmol/l of respective primers, 0.25 μmol/l of respective probes and 10 ng of DNA in a final volume of 20 μl. Droplets were generated using automated droplet generator (Bio-Rad catalog #186-4101) following the manufacturer’s protocol. PCR parameters for *LBR* locus were 10 s at 95°C, then 40 cycles of 94°C for 30 s, 60°C for 30 s and 72°C for 2 min followed by 98°C for 10 min with a ramping of 2°C/s at all steps. The PCR cycling parameters for *ESR1* genomic locus were 10 s at 95°C, then 40 cycles of 94°C for 30 s and 60°C for 1 min followed by 98°C for 10 min with a ramping of 2°C/s at all steps. After PCR amplification, droplet reader (Bio-Rad QX200™ Droplet Reader Catalog #1864003) was used to measure the end-point fluorescence signal in droplets as per the manufacturer’s protocol. The recorded data were subsequently analyzed with QuantaSoft software version 1.7.4.0917 (Bio-Rad). Each TaqMan probe was evaluated for sensitivity and specificity.

## RESULTS

### p53-deficient cells exhibit radioresistance and accelerated resolution of DNA DSBs

**Figure 1.**
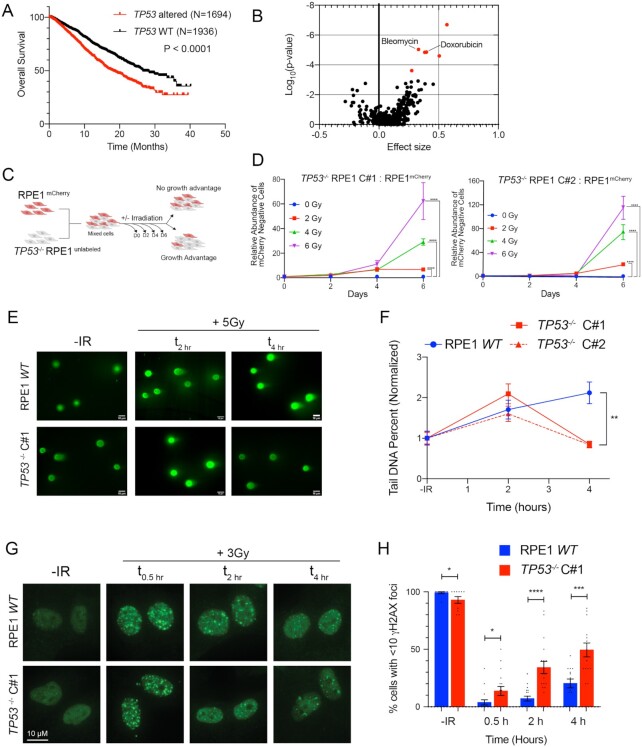
p53-deficient cells exhibit radioresistance and accelerated resolution of DNA DSBs. (**A**) Kaplan–Meier overall survival analysis of patients from the MSK-IMPACT pan-cancer cohort, stratified by genetic alterations in the *TP53* gene (WT vs. mutations or deep deletions). Data obtained from the cBioportal. *P*-value calculated using a two-sided log-rank test. (**B**) Association between *TP53* mutation and drug sensitivity from the Genomics of Drug Sensitivity in Cancer database. *P*-values were calculated using ANOVA and a threshold of *P* < 10^−3^ and a false discovery rate threshold of 25% were used to indicate statistically significant associations (labeled in red). (**C**) Diagram of growth competition assay. mCherry-labeled RPE1 cells were mixed with unlabeled *TP53*^−/−^ RPE1 (1:1), exposed to IR and grown for 6 days. (**D**) Relative abundance of unlabeled *TP53*^−/−^ Clone#1 (left panel) or *TP53*^−/−^ Clone#2 (right panel) measured by Intellicyte high-throughput cytometry ± standard error of the mean (SEM, *n* = 6) is shown, normalized to the untreated (0 Gy) cohort at each time point. (**E**) Representative neutral COMET fluorescence staining for measurement of DNA DSBs in cells with indicated genotypes treated without or with 5 Gy IR evaluated at the indicated time points. (**F**) Quantification of neutral COMET tail DNA percentage, normalized to the untreated baseline, in RPE1 *WT* (blue) and two *TP53*^−/−^ RPE1 cell lines (red). Data shown are mean values (*n* = 50–150 cells per treatment condition) ± SEM, and are consistent across three independent biological replicates. (**G**) Representative immunofluorescence images of γH2AX foci in cells with indicated genotypes untreated (no IR) or collected 0.5, 2 and 4 h after IR (3 Gy). (**H**) Percentage of cells with <10 γH2AX foci at the different time points in RPE1 *WT* (blue) and *TP53^−^^/^^−^* RPE1 (red). Data shown are mean ± SEM across three independent biological replicates. *P < 0.05, **P < 0.01, ***P < 0.001 and ****P < 0.0001 by two-tailed Student’s *t*-test.

We used the MSK-IMPACT pan-cancer cohort to investigate the prevalence and prognostic impact of TP53 mutations on the survival of patients with metastatic cancer (32). Out of 4732 evaluable cases, 46% harbored *TP53* gene alteration and these patients had significantly worse overall survival than patients without somatic *TP53* gene alteration [Figure 1A, HR = 1.612 (95% CI 1.441–1.803), *P* = 1.1 × 10^−16^]. While there are several potential explanations for this observation, we postulated that this effect may at least in part be due to increased resistance to cancer therapy in the setting of p53 deficiency. Thus, we queried the Genomics of Drug Sensitivity in Cancer database to investigate a potential genetic association between p53 deficiency and therapeutic resistance (33). Across over 600 different cancer cell line models and ∼350 therapeutic compounds, there was a significant correlation between *TP53* mutation status and increased resistance to the DNA DSB-inducing chemotherapeutic agents, bleomycin and doxorubicin (Figure 1B, bleomycin, *P* = 9.2 × 10^−6^, and doxorubicin, *P* = 1.4 × 10^−5^). To further establish whether p53 deficiency is sufficient to induce resistance to clastogenic therapy, we used CRISPR/Cas9 to disrupt *TP53* in the p53-proficient immortalized epithelial cell line model hTERT-RPE1 (‘RPE1’), which is a preferred model for investigating p53-dependent cell fate (34–36). Two independent CRISPR/Cas9-targeted *TP53^−^^/^^−^* RPE1 clones were selected for further study after confirming cells were deficient for p53 protein and lacked p53-dependent transcriptional induction of *CDKN2A* (p21 WAF1/CIP1) in response to ionizing radiation (IR) (Supplementary Figure S1A–C).

To assess whether p53 deficiency confers a proliferative advantage when treated with IR, we performed a mixed competition assay. We mixed mCherry-labeled RPE1 with equal numbers of unlabeled *TP53^−^^/^^−^* RPE1 or p53-proficient RPE1 (control) (Figure 1C). We quantified the relative abundance of the unlabeled cells after exposure to IR (0–6 Gy), normalized to untreated samples at each time point. RPE1-labeled and unlabeled cells maintained stable representation across time and treatment conditions (Supplementary Figure S1D). Additionally, p53-deficient cells did not demonstrate a proliferation advantage in the absence of IR. However, treatment with IR at any dose level led to statistically significant positive selection for p53-deficient cells (Figure 1D). Similar effects were also observed by the clonogenic survival assay after IR and the radiomimetic NCS (Supplementary Figure S1E and F). Thus, p53 deficiency in this isogenic model is sufficient to induce radioresistance.

Unrepaired DSBs can suppress proliferation through the engagement of DNA damage-induced cell cycle checkpoints. We examined kinetics of IR-induced DSB repair in p53 WT and two independent *TP53^−^^/^^−^* RPE1 clones by performing neutral COMET analysis (Figure 1E and F). Despite similar peaks of IR-induced DSBs, p53-deficient cells exhibited accelerated resolution of DSBs by 4 h post-IR. Concordantly, the percentage of cells with <10 γH2AX foci was significantly higher in *TP53^−^^/^^−^* cells (Figure 1G and H). Based on these observations, we postulated that radioresistance induced by p53 deficiency in the RPE1 immortalized cell line model is associated with accelerated DSB repair.

### Partial inhibition of DNA-PK restores DNA damage foci formation in p53-deficient cells

**Figure 2.**
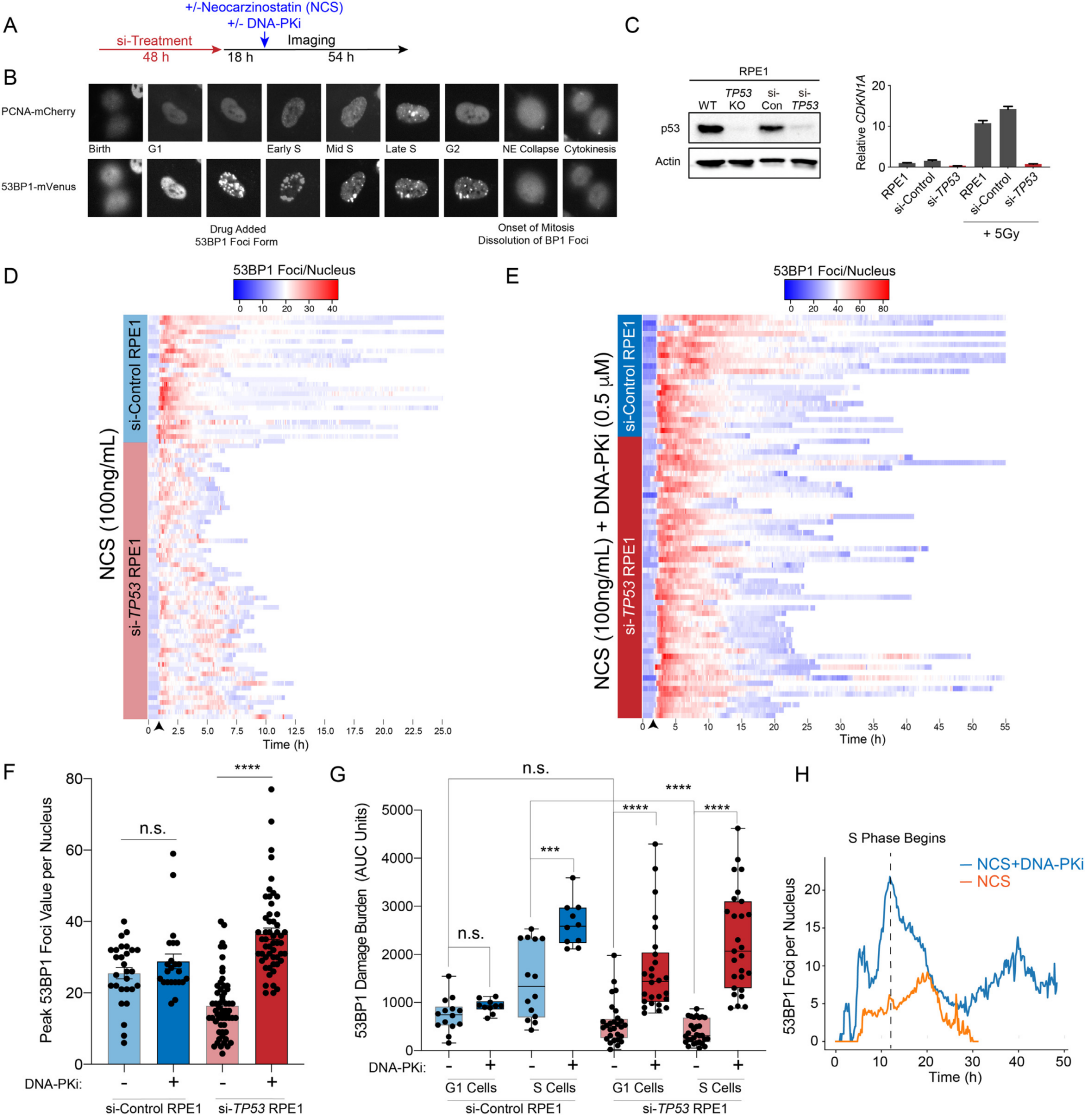
Inhibition of DNA-PK restores DNA damage foci formation in p53-deficient cells. (**A**) Live cell imaging procedure. Cells transfected twice with 10 nM si-Control or si-*TP53* for 48 h prior to imaging. Eighteen hours into imaging, cells are treated with NCS (100 nM), DNA-PKi (0.5 μM NU7441) or both and imaged for a total of 72 h. (**B**) RPE1 cells expressing the PCNA-mCherry and 53BP1-mVenus reporters. Cell cycle phases delineated by PCNA foci and DNA DSBs are marked by 53BP1 foci. (**C**) Validation of si-TP53 efficacy by immunoblotting for p53 (left panel) and IR (5 Gy) induced *CDKN1A* mRNA levels (right panel). (**D**) Heatmap of 53BP1 foci tracings for single cells tracked from birth to mitosis or end of imaging: for si-Control (*n* = 30 cells) and si-*TP53*-treated RPE1 (*n* = 60 cells) treated with NCS 100 ng/ml. For visualization, cells are aligned to 10 frames prior to drug addition (black arrow). (**E**) Heatmap of 53BP1 foci tracings for si-Control (*n* = 25 cells) and si-*TP53*-treated RPE1 cells (*n* = 55 cells) treated with 100 ng/ml NCS + 0.5 μM DNA-PKi (NU7441). (**F**) Peak 53BP1 foci counts for cells treated with 100 ng/ml NCS or NCS + 0.5 μM DNA-PKi. Shown are the mean ± SEM. Significance determined using two-tailed *t*-test. (**G**) Area under the curve (AUC) analysis of 53BP1 burden showing integral DNA damage for cells treated with NCS versus NCS and DNA-PKi. Cells are segregated into two groups: cells exposed to drug in G1 versus S phase (*n* = 25–30 G1 or S cells for si-*TP53* cohort; *n* = 10–15 G1 or S cells for si-Control cohort). Significance determined by two-tailed *t*-test: ****P < 0.0001, ***P < 0.001 and n.s. = non-significant. (**H**) Kinetic analysis of 53BP1 foci burden in G1-phase p53-deficient RPE1 upon exposure to NCS without (orange line) or with (blue line) concomitant DNA-PKi (*n* = 30 cells for each condition). Dashed line = S-phase onset.

To directly assess the relationship between DSB repair kinetics, cell cycle status and cell fate at the single-cell level, we established a live cell imaging platform (Figure 2A). RPE1 cells were dually labeled with PCNA-mCherry (to monitor cell cycle state transitions) and 53BP1-mVenus (to monitor DSB foci kinetics) (Figure 2B) (34,37). These dual-labeled cells were treated with scrambled siRNA (si-Control) or siRNA targeting *TP53* (si-*TP53*), the latter of which resulted in >90% knockdown of *TP53* transcript and protein, as well as elimination of p53-dependent *CDKN1A* transcription in response to IR (Figure 2C). Forty-eight hours after siRNA treatment, RPE1 cells were imaged for a total of 72 h every 10 min, and 18 h into imaging, the radiomimetic NCS (100 ng/ml) was added (Figure 2A). NCS has been previously utilized in live cell imaging studies and has been shown to induce peak DSBs within 10 min of drug addition. This experimental design allowed us to determine the cell cycle status of each cell within the asynchronous cell population at the time of NCS exposure. After NCS treatment, single-cell analyses for DSB repair foci kinetics and cell cycle outcomes were performed. Consistent with colony forming assays depicting resistance to NCS induced by TP53 deficiency (Supplementary Figures S1F and S2C and D), analysis of global proliferation by live cell imaging revealed significantly greater proliferation of p53-deficient RPE1 cells relative to controls after NCS treatment (Supplementary Figure S2A and B). Of note, si-TP53 did not alter the basal cell cycle profile of untreated RPE1 cells (Supplementary Figure S2E and F).

To analyze DSB repair kinetics in cells exposed to NCS, we tracked and quantified 53BP1 foci in single cells and plotted heatmaps of damage foci burden over time from cell birth to mitosis (Figure 2D). Our results indicate that cells with functional p53 sustain high levels of damage foci in a prolonged manner after NCS exposure. In contrast, p53-deficient cells on average developed a lower peak burden of 53BP1 foci after NCS treatment, with accelerated resolution of damage foci to baseline levels (Figure 2D and F). Moreover, p53-deficient cells also exhibited greater variance in 53BP1 foci burden and kinetics. Given the rapidity with which 53BP1 foci were being resolved, we hypothesized that NHEJ repair may be mediating this effect. We thus performed the same experiment in the presence of a DNA-PKi (NU7441), which targets the central kinase in the NHEJ pathway (38,39). To minimize the possibility of off-target effects, and to better mimic clinically achievable levels of pathway inhibition, we selected a low dose of DNA-PKi (0.5 μM NU7441) that resulted in 50% inhibition of IR-induced DNA-PK phosphorylation (Supplementary Figure S2G). Strikingly, low-dose DNA-PKi qualitatively abolished the difference in 53BP1 kinetics after NCS treatment between p53-deficient and p53-proficient cells (Figure 2E). To quantitatively assess the magnitude of difference in damage burden, we calculated peak maximum 53BP1 foci values for each cell represented in the heatmap (Figure 2F). Consistent with the heatmap representation, the mean peak foci count after NCS treatment was 40% lower in si-*TP53*-treated cells relative to controls (Figure 2F, *P* < 0.0001). Notably, DNA-PKi treatment resulted in a >2-fold increase in peak 53BP1 foci levels in the p53-deficient cells, whereas there was no significant effect in control cells (Figure 2F). These results indicate that DNA-PK activity is required for accelerated resolution of radiomimetic-induced DNA damage foci in p53-deficient cells.

We next evaluated whether the effects of p53 deficiency and DNA-PKi on DNA damage burden are influenced by cell cycle status at the time of drug exposure. We used PCNA live cell imaging to resolve cell cycle phase transitions in cells tracked for 53BP1 foci kinetics. AUC analyses were performed in single cells to estimate total DNA damage burden during G1 and S phases after NCS exposure (Figure 2G). This analysis revealed that the diminished 53BP1 foci burden observed in p53-deficient cells was most pronounced during S phase relative to control cells (Figure 2G). DNA-PKi treatment significantly increased S-phase 53BP1 burden in both si-Control and si-*TP53*-treated RPE1 cells (Figure 2G). While si-*TP53*-treated cells in G1 had similar 53BP1 foci burden compared to si-Control cells, DNA-PKi treatment selectively increased damage burden in si-TP53 cells exposed in G1. We hypothesized that this effect may be due to loss of the p53-dependent G1/S checkpoint resulting in propagation of unrepaired DNA damage into S phase. Indeed, we found that DNA-PKi induced a drastic increase in 53BP1 foci as p53-deficient cells transitioned from G1 to S phase, which subsequently diminished over time (Figure 2H, *P* < 0.00001 at *t* = start of S phase). Thus, DNA-PK is required for resolution of clastogen-induced DSB foci in p53-deficient cells, and most prominently during S phase.

### Impaired DNA damage-induced cell cycle checkpoint responses in p53-deficient cells are partially restored by DNA-PKi

**Figure 3.**
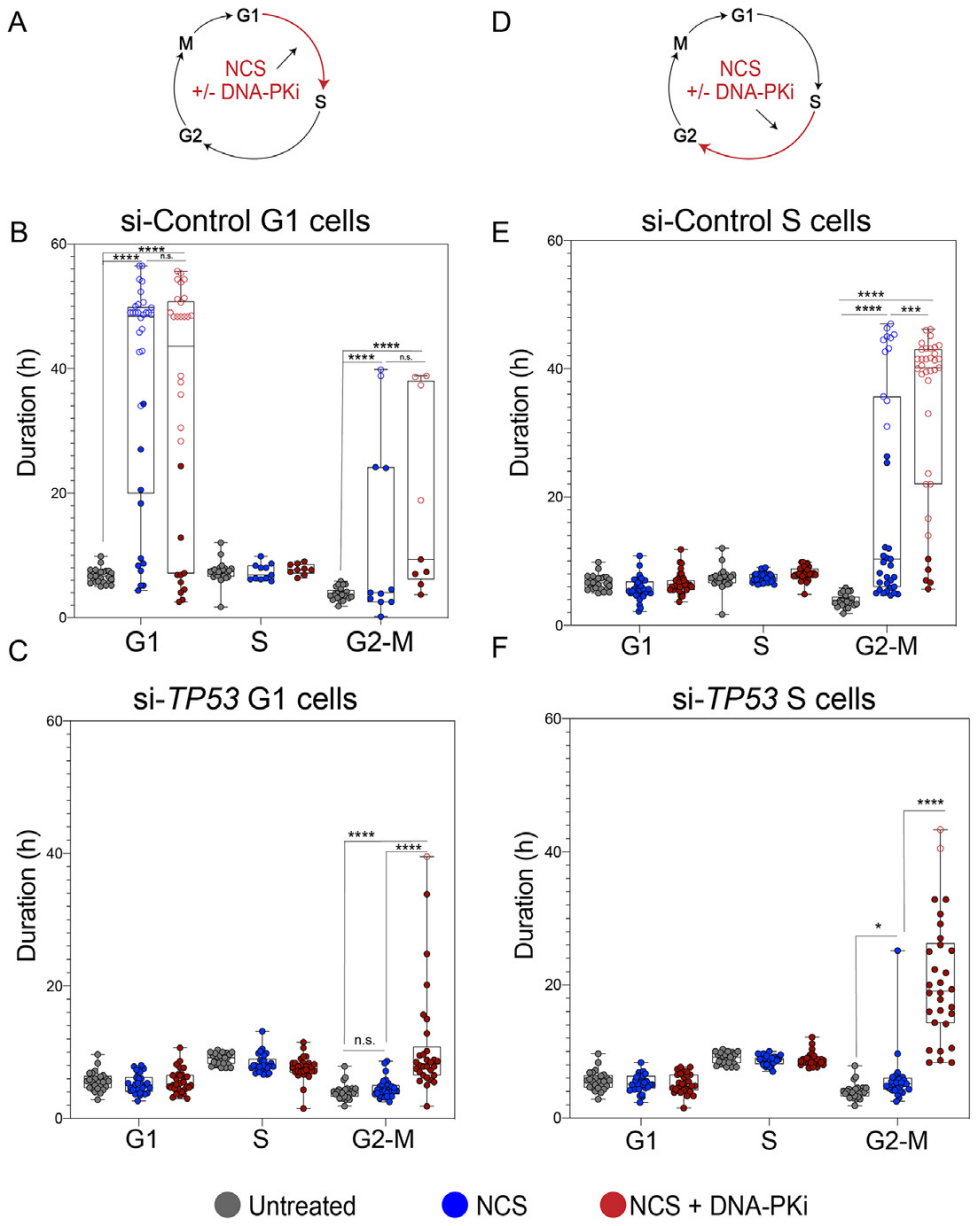
Checkpoint responses halt p53-proficient cells upon exposure to NCS while p53-deficient cells continue to cell cycle despite NCS exposure. (**A**) Schematic depicting NCS treatment (100 ng/ml) and/or NCS + 0.5 μM DNA-PKi (NU7441) treatment, and phase of the cell cycle cells exposed to drug (G1). (**B**) Distribution of cell cycle phase lengths; each colored dot is an individual cell with untreated cells (no NCS) shown in gray, NCS-treated cells shown in blue and NCS + 0.5 μM DNA-PKi-treated cells shown in red for si-Control RPE1 in G1 phase. *n* = 20 untreated and *n* = 30 treated cells (for each treatment cohort). Statistical significance was determined by comparing untreated and treated groups at each phase: ****P < 0.0001 and n.s. = non-significant. Open circles indicate arrested cells that did not enter the subsequent phase of cell cycle for remainder of imaging. (**C**) Distribution of cell cycle phase lengths for si-*TP53*-treated RPE1 in G1 phase: ****P < 0.0001 and n.s. = non-significant as evaluated by two-tailed *t*-test. (**D**) Schematic of drug treatment for cells in S phase. (**E**) Distribution of cell cycle phase lengths for si-Control treated RPE1 in S phase: ***P < 0.001, ****P < 0.0001 and n.s. = non-significant as evaluated by two-tailed *t*-test. (**F**) Distribution of cell cycle phase lengths for si-*TP53*-treated RPE1 in S phase: ****P < 0.0001 and n.s. = non-significant as evaluated by two-tailed *t*-test.

To investigate the association between DNA damage and activation of cell cycle checkpoints, we quantified cell cycle phase durations for all treatment conditions (Figure 3A and D). Time-lapse microscopy of live cells expressing the PCNA biosensor enables us to deconvolute biological effects in different cell cycle stages without the use of any synchronization agents. Specifically, we separately evaluated the effect of NCS without and with DNA-PKi treatment on cells that were in either G1 or S phase at the time of drug exposure (Figure 3A and D). p53-proficient G1 cells exposed to NCS induced a significant prolongation of G1, indicative of G1/S checkpoint activation, with a substantial proportion of cells remaining arrested for the duration of imaging (Figure 3B). Similarly, cells exposed to NCS in S phase exhibited a G2–M checkpoint (Figure 3E). p53-deficient cells exhibited no prolongation of G1 duration after NCS, consistent with the notion that G1/S checkpoint activation is p53 dependent (Figure 3C) (40,41). Similarly, p53-deficient cells also displayed a defective G2/M checkpoint. DNA-PK inhibition did not alter G1 duration in either p53-proficient or p53-deficient cells (Figure 3B and C). In contrast, DNA-PKi increased the duration of time spent in G2–M phase irrespective of p53 status (Figure 3E and F). These observations suggest that increased levels of S-phase DNA damage induced by DNA-PKi and NCS treatment (see Figure 2G and H) result in enhanced activation of a G2/M checkpoint that is, at least partially, p53 independent. However, the duration of G2/M arrest differed by p53 status. While p53-proficient cells frequently remained arrested for the entire duration of imaging (open circles, Figure 3B and E), p53-deficient cells experienced a more transient prolongation of G2 duration followed by progression into mitosis (Figure 3C and F).

### Inhibition of DNA-PK induces catastrophic mitoses in p53-deficient cells

**Figure 4.**
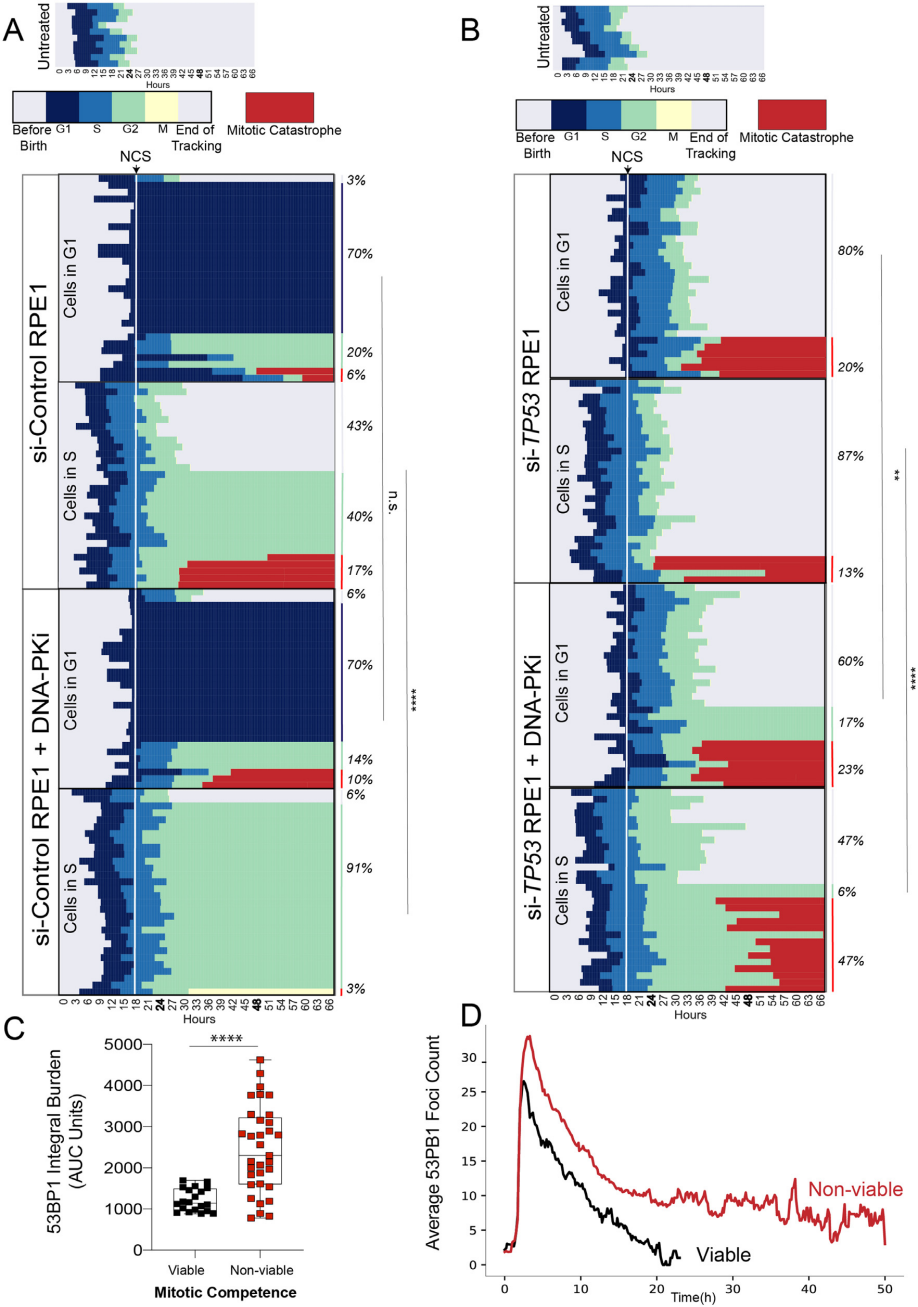
Inhibition of DNA-PK induces catastrophic mitoses in p53-deficient cells. Horizontal bar plots depicting cell cycle outcomes for si-Control (**A**) and si-*TP53* (**B**) treated RPE1 cells. Colored bars indicate different phases of the cell cycle; legend shown with no treatment control for comparison. Cells with red bars at the end of mitosis indicate terminal cell cycle event (mitotic catastrophe or apoptosis). The horizontal axis indicates time point during imaging (hours). Individual cells are tracked from birth to completion of mitosis or end of imaging. The upper panels depict cell cycle outcomes for untreated cells. The lower panels depict cells treated with drug (100 ng/ml NCS or 100 ng/ml NCS + 0.5 μM DNA-PKi), with time of drug addition denoted with a white line. Each row is an individual cell (*n* = 60 cells for each condition). Cells are organized based on cell cycle state at the time of drug addition (G1 versus S), and cell fate outcomes. Event frequency is reported as a percentage on the right. Fisher’s exact two-tailed test was performed between −/+ DNA-PKi cohorts using two outcome groups [viable versus non-viable (arrested cells + mitotic catastrophe outcomes)]: ****P < 0.0001 and n.s. = non-significant. (**C**) AUC analysis of 53BP1 integral damage burden in viable versus non-viable p53-deficient cells that were treated with NCS and DNA-PKi. Statistical significance was calculated using a two-tailed Mann–Whitney test comparing ranks: ****P < 0.0001. (**D**) Kinetic analysis of 53BP1 foci burden over time in p53-deficient RPE1 segregated by mitotic viability. The red line corresponds to mean 53BP1 foci burden over time for all p53-deficient cells treated with NCS and DNA-PKi that undergo catastrophic mitoses; black line indicates mean foci burden over time for p53-deficient cells with NCS and DNA-PKi treatment that are viable post-mitosis (*n* = 20 viable cells and *n* = 33 non-viable cells).

We next used a bar graph representation to track the fate of individual cells from birth until mitosis (Figure 4A and B, top panels). Red bars indicate a mitotic catastrophe or apoptosis event (Supplementary Figure S3A and B). The median cell cycle time for both untreated p53-proficient and p53-deficient cells was ∼22–24 h. NCS treatment is indicated as a dashed line at the 18 h time point. Individual cells are ordered according to cell cycle phase at the time of NCS treatment (G1 versus S) and eventual cell fate (viable, G1 arrest, G2 arrest or mitotic catastrophe/apoptosis). The majority (70%) of p53-proficient (si-Control) G1 cells exposed to NCS activated a G1 checkpoint that was maintained for the remainder of imaging (Figure 4A). 26% of these cells underwent G2 arrest or mitotic catastrophe, whereas only 3% retained their proliferative capacity (Figure 4A). Control cells exposed to NCS in S phase exhibited more diverse cell fates: 40% G2 arrest, 17% mitotic catastrophe and 43% retained proliferative capacity. These observations, made using single-cell tracking of asynchronous cell populations, are consistent with observations of intrinsic radioresistance of S-phase cells using cell synchronization methods (42). In contrast, the majority of p53-deficient (i.e. si-*TP53*-treated) cells in G1 or S phase at the time of NCS treatment remained viable without perceptible engagement of any cell cycle checkpoints (Figure 4B, 80% and 87%, respectively). Consistent with prior 53BP1 analyses, S-phase cells are most sensitized to DNA-PKi, with an increased frequency of prolonged G2 arrest in control cells from 40 to 91%, and increased mitotic catastrophe in p53-deficient cells from 13 to 47%, (Figure 4A and B). In total, the percentage of viable p53-deficient cells after NCS treatment in S phase decreased from 87% to 47% with DNA-PK inhibition (*P* < 0.0001, Fisher’s exact test).

Despite the significant increase in mitotic catastrophe induced by combined treatment with DNA-PKi and NCS, 47% of p53-deficient cells exhibited intrinsic resistance to therapy and retained proliferative viability, compared to only 6% of p53-proficient cells (Figure 4B). We postulated that the level of unrepaired DNA damage within individual cells may correlate with the observed viable (i.e. resistant) versus non-viable (i.e. sensitive) cell fates. To evaluate this hypothesis, we quantified integral DNA damage burden in p53-deficient RPE1 cells with viable versus non-viable mitotic outcomes (Figure 4C). The mean integral DNA damage burden was ∼2-fold higher in non-viable cells, relative to cells that viably completed mitosis (*P* < 0.0001). Further analysis revealed that integral DNA damage burden in S phase was most highly associated with cell viability after drug treatment (Supplementary Figure S3C). In addition, we tracked the average 53BP1 foci burden over time for these two cohorts (Figure 4D). Our results indicate that cells with non-viable mitotic outcomes have an increased peak value of DNA damage after treatment with DNA-PKi and NCS, which remains elevated over time (*P* < 0.0001 at *t* = 20 h, Figure 4D). Conversely, p53-deficient cells that retained viability after treatment with NCS and DNA-PKi exhibited increased proficiency in DNA damage repair resulting in lower foci burden, raising the possibility that intrinsic therapeutic resistance may be due to the presence of compensatory DSB repair pathways.

### Pol θ-mediated end joining repair promotes viability of p53-deficient cells

**Figure 5.**
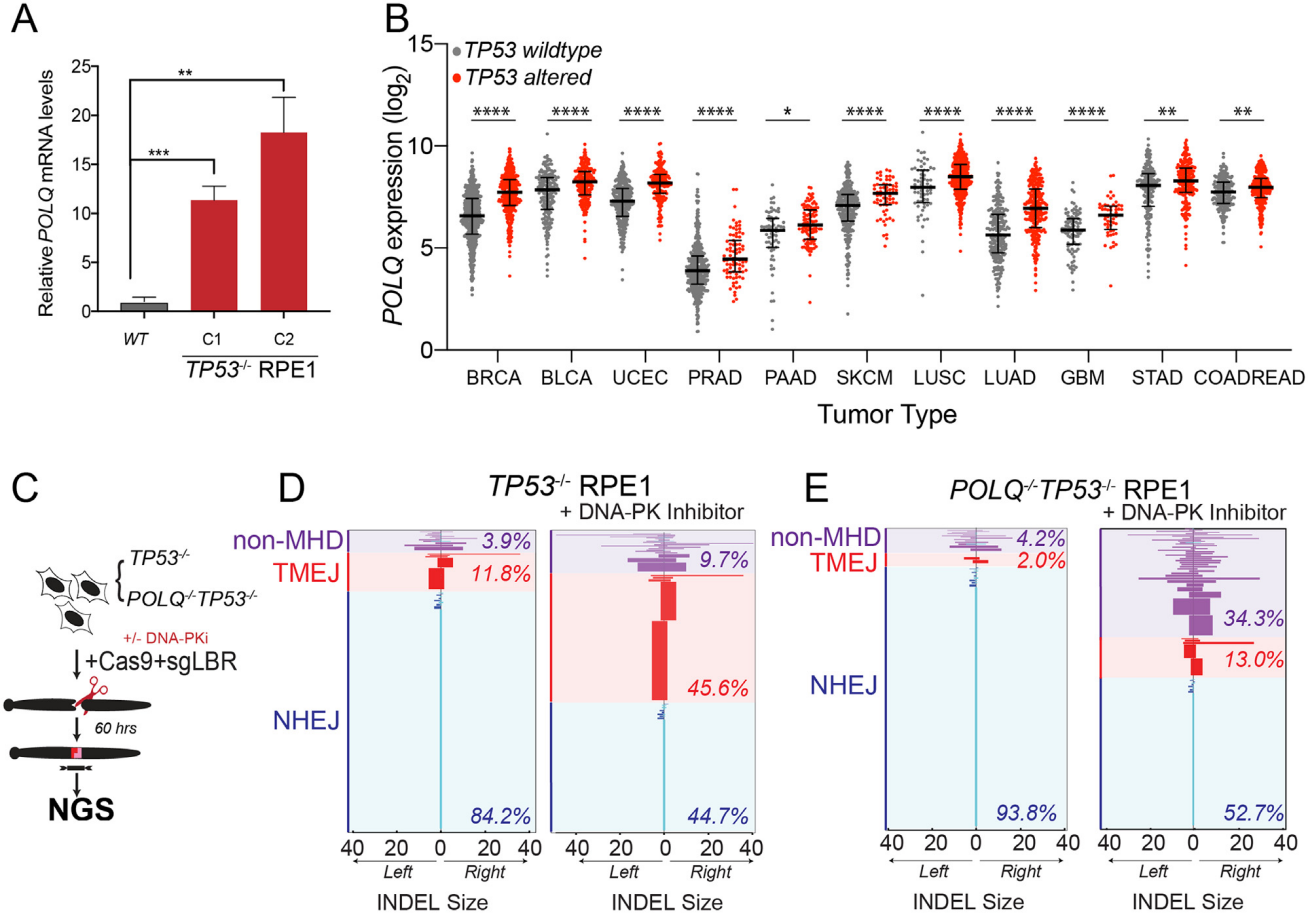
p53-deficient cells utilize alternative end joining pathways in the absence of active DNA-PK. (**A**) RT-qPCR for *POLQ* mRNA levels in two *TP53*^−/−^ RPE1 clones compared to WT RPE1. Significance was determined using two-tailed *t*-test: ****P < 0.0001 and **P < 0.01. (**B**) *POLQ* gene expression depicted as log_2_ values of *TP53* wild-type versus mutant cancers across a subset of TCGA tumor types. Tumor labels follow TCGA labeling format. BRCA: breast cancer; BLCA: B-cell lymphoma; UCEC: uterine cancer; PRAD: prostate cancer; PAAD: pancreatic cancer; SKCM: melanoma; LUSC: lung squamous cell cancer; LUAD: lung adenocarcinoma; GBM: glioblastoma multiforme; STAD: stomach cancer; COADREAD: colorectal cancer. ****P < 0.0001, ***P < 0.001, **P < 0.01 and *P < 0.05, as calculated by one-way ANOVA. (**C**) Schematic depicting chromosomal break repair assay. *TP53*^−/−^ and *POLQ^−^^/^^−^TP53*^−/−^ RPE1 are segregated into two cohorts (±3 μM DNA-PKi). Cells are electroporated using Cas9-RNP-sgRNA-*LBR* and evaluated by amplicon next-generation sequencing (NGS) for break repair products at target locus. (**D**) Horizontal bar chart representation of individual break repair products at *LBR* locus in (**D**) *TP53*^−/−^ RPE1 and (**E**) *TP53^−^^/^^−^POLQ^−^^/^^−^* RPE1 by NGS. Position 0 denotes *LBR* locus cut site, with left and right positions denoting final INDEL size and orientation. Data shown represent an average of three biological replicates.

Prior studies have demonstrated that cells with NHEJ deficiency exhibit a compensatory increase in alternative end joining repair mediated by Pol θ (gene *POLQ*) (29,43,44). TMEJ of DNA DSBs is characterized by deletions and templated insertions that are flanked by short tracts of sequence identity, or microhomology (29,45). We found that *POLQ* mRNA expression was 10–20-fold higher in two independent *TP53^−^^/^^−^* RPE1 clones, relative to parental *TP53* wild-type cells (Figure 5A). In contrast, acute knockdown of *TP53* only resulted in a 20% increase in *POLQ* expression, suggesting that the observed effect in stable p53-deficient cells is predominantly indirect (Supplementary Figure S4A). Using the publicly available pan-cancer TCGA dataset, we also observed a correlation between *POLQ* mRNA overexpression and *TP53* mutation status in breast, lung, bladder, colorectal, gastric, glioblastoma, pancreatic, prostate, melanoma and uterine cancers (Figure 5B) (46–48). Prior studies have also observed an association between *POLQ* overexpression in cancer with *BRCA1*/*BRCA2* mutation and an over-representation of TMEJ-associated genomic scars (43,45,49). To assess whether *POLQ* overexpression contributes to therapeutic resistance of *TP53^−^^/^^−^* RPE1 cells to NCS and DNA-PKi, we validated *POLQ* knockdown efficiency by siRNA targeting, as well as inhibition of TMEJ repair using an extrachromosomal substrate assay (Supplementary Figure S4B and C) (29). Additionally, we generated a double knockout *POLQ^−^^/^^−^TP53^−^^/^^−^* RPE1 line by CRISPR targeting (Supplementary Figure S4D). Biallelic frameshift mutations in *POLQ* were confirmed by Sanger sequencing, which was also associated with deficiency in TMEJ repair (Supplementary Figure S4E) (29).

To directly assess whether TMEJ repair is increased after DNA-PKi treatment in p53-deficient RPE1 cells, we analyzed chromosomal break repair patterns at a site-specific chromosomal DSB. Cells were transfected with Cas9 ribonucleoprotein (RNP) complexes that target the *LBR* locus, with or without DNA-PKi. Genomic DNA was harvested 60 h later and analyzed for break repair patterns using NGS (Figure 5C). Target amplification and TIDE analyses confirmed high rates of target site cleavage in all samples transfected with a full complement of Cas9-RNP (Supplementary Figure S4F and G) (50). We applied a bioinformatic algorithm to characterize the spectrum of repair products with at least 0.1% prevalence, classified according to the size of left deletion, right deletion, insertion and microhomology (ScarMapper, see the ‘Materials and Methods’ section) (43). INDELs <5 bp were categorized as NHEJ, with the predominant repair product being a +A 1 bp insertion (51). TMEJ was defined as repair products whose frequency was diminished by at least 2-fold in *POLQ^−^^/^^−^* cells. All other repair products were categorized as ‘unclassified’. DNA-PK inhibition in *TP53^−^^/^^−^* RPE1 cells resulted in a substantial reduction in NHEJ repair (84% to 45%), with a compensatory increase in TMEJ to nearly 46% (from 12%) of all DSB repair (Figure 5D). In contrast, DNA-PK inhibition in *POLQ^−^^/^^−^TP53^−^^/^^−^* RPE1 cells resulted in relatively lower levels of TMEJ signature repair (13%), and increased levels of unclassified repair products (34.3%, versus 9.7% in *TP53^−^^/^^−^* cells) (Figure 5E). A limitation of NGS analysis of DSB break repair is that non-amplifiable target loci are not measured. Thus, we used digital PCR to quantify the *LBR* locus detection rate, relative to a control locus, upon inhibition of DNA-PK and/or Pol θ (Supplementary Figure S4H). *LBR* locus detection rates were most reduced upon dual inhibition of DNA-PK and Pol θ, indicating an increase in unrepaired DSBs upon inhibition of both DSB repair pathways (Supplementary Figure S4H). These observations confirm an essential role for TMEJ in compensatory repair of chromosomal DSBs upon pharmacological inhibition of DNA-PK.

**Figure 6.**
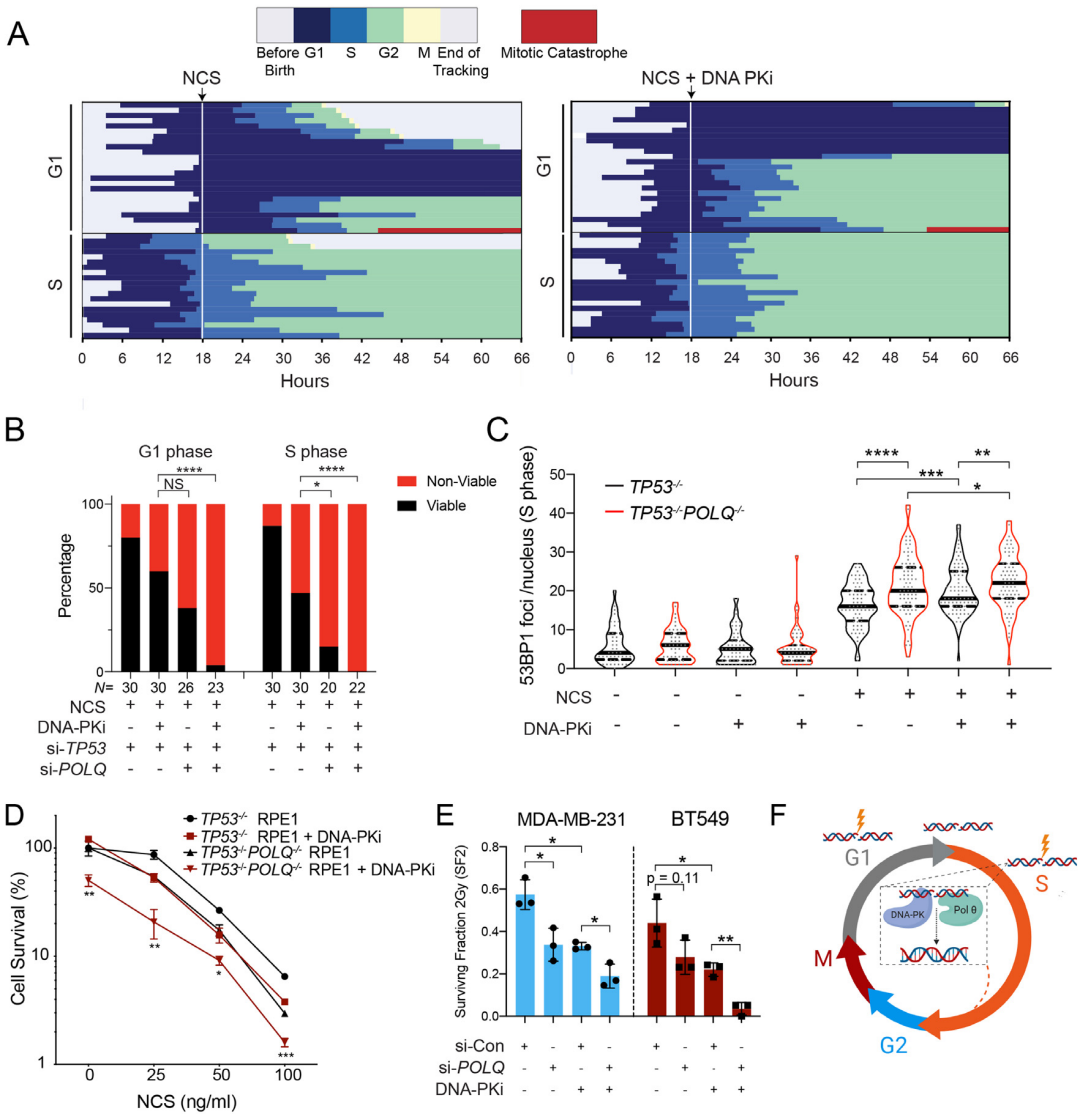
Pol θ promotes resistance to radiomimetic therapy in p53-deficient cells by suppressing S-phase DSBs. (**A**) Horizontal bar plots depicting cell cycle outcomes for si-*TP53* + *POLQ* RPE1 cells treated with 100 ng/ml NCS (left panel) or 100 ng/ml NCS plus 0.5 μM DNA-PKi (NU7441) (right panel). Each row represents an individual cell, and the cells are organized according to cell cycle state at the time of drug addition (G1 versus S). (**B**) Analysis of cell fate outcomes categorized as viable (i.e. successful completion of mitosis) versus non-viable (arrest or mitotic catastrophe). Fisher’s exact two-tailed test: *P < 0.05 and ****P < 0.0001. (**C**) Violin plots of 53BP1 foci counts per nucleus in *TP53^−^^/^^−^* (black) and *TP53^−^^/^^−^POLQ^−^^/^^−^* (red) RPE1 cells, 4 h after treatment with 100 ng/ml NCS with or without 0.5 μM DNA-PKi. Data shown are for S-phase cells, which were identified by a 30-min EdU pulse at the time of drug addition. The violin plots show the median (solid black line) and quartiles (dashed lines). Statistical significance assessed with Student’s two-tailed *t*-test: ***P < 0.001 and ****P < 0.0001. (**D**) Colony forming efficiency assay evaluating *TP53*^−/−^ and *POLQ^−^^/^^−^TP53*^−/−^ RPE1 after treatment with NCS (at 25, 50 and 100 ng/ml) with or without 0.5 μM DNA-PKi. Cell survival is normalized to the untreated control for each genotype, and the bar graph depicts mean ± SEM (*n* = 3). Statistical significance of *TP53^−^^/^^−^* versus *TP53^−^^/^^−^POLQ^−^^/^^−^* genotypes assessed with Student’s two-tailed *t*-test: ***P < 0.001, **P < 0.01 and *P < 0.05. (**E**) Surviving fraction after 2 Gy IR as determined by the colony forming assay in MDA-MB-231 (left) and BT-549 (right) *TP53*-mutant breast cancer cell line models. Cells were pretreated with si-Control or si-*POLQ* for 48 h prior to irradiation without or with DNA-PKi (0.5 μM NU7441). Statistical significance assessed with Student’s two-tailed *t*-test: *P < 0.05 and **P < 0.01. (**F**) DNA-PK and Pol θ independently promote repair of therapy-induced S-phase DNA DSBs, which promotes therapeutic resistance in p53-deficient cells.

To determine the impact of *POLQ* inhibition on cell fate after NCS exposure in p53-deficient RPE1 cells, we performed live cell imaging in p53-deficient (i.e. si-*TP53*) versus dual p53/Pol θ-deficient (i.e. si-*TP53* and si-*POLQ*) cells. Strikingly, p53-deficient cells with *POLQ* inhibition exhibited significantly lower levels of proliferative viability after treatment with NCS—38% for cells treated in G1 phase and 15% for cells treated in S phase (Figure 6A and B). Viability was further reduced when combined with low-dose DNA-PK inhibition—4% for cells treated in G1 phase and 0% for cells treated in S phase. *POLQ* inhibition also resulted in a distinct profile of therapeutic response in p53-deficient cells, favoring G2 and G1 checkpoint activation rather than induction of mitotic catastrophe. Thus, Pol θ inhibition during NCS treatment results in robust activation of p53-independent cell cycle checkpoint pathways. We next evaluated the impact of *POLQ* inhibition on NCS-induced DSB burden, and found significantly increased 53BP1 foci in *TP53^−^^/^^−^POLQ^−^^/^^−^* RPE1 cells relative to parental *TP53^−^^/^^−^* RPE1 cells (Figure 6C). The increase in DSB burden in *POLQ*-deficient cells was most pronounced in S phase, and no additional difference was observed when all cells were analyzed (Supplementary Figure S5A and B).

Consistent with the profound effects of Pol θ inhibition on NCS-induced DSB burden and cell fate, we observed significantly reduced clonogenic survival of *POLQ^−^^/^^−^TP53^−^^/^^−^* RPE1 lines after treatment with NCS, with or without low-dose DNA-PKi (Figure 6D). Notably, the effects of DNA-PK and Pol θ inhibition were additive, consistent with independent roles in promoting survival after DSB-inducing therapy. To evaluate the relevance of these findings to cancer, we investigated the p53-mutant breast cancer cell line models MDA-MB-231 and BT549, both of which represent the ‘triple-negative’ subtype of breast cancer. Analogous to the RPE1 experiments, we used low-dose DNA-PKi (0.5 μM NU7441) and si-*POLQ* to inhibit NHEJ and TMEJ, respectively (Supplementary Figure S5C). Consistent with our observations in the RPE1 model, we observed additive effects of DNA-PK and Pol θ inhibition in reducing the surviving fraction at 2 Gy (SF2), measured by colony forming survival assays (Figure 6E). Collectively, these findings establish TMEJ as an independent repair pathway that reduces radiomimetic-induced S-phase DNA damage and limits the efficacy of DNA-PK inhibition.

## DISCUSSION

*TP53* gene alterations are strongly associated with resistance to DSB-inducing cancer therapies and shortened survival of cancer patients. Thus, there is a critical need to develop strategies to overcome therapeutic resistance mediated by p53 deficiency. This study provides a detailed analysis of how p53 deficiency alters the kinetics of DSB repair after clastogenic therapy, as well as the relationship between therapeutic DSBs and cell cycle outcomes. Our study reveals independent roles for DNA-PK and Pol θ in repairing S-phase DSBs after radiomimetic treatment in p53-deficient cells, which collectively mediate radiation resistance (Figure 6F). Accordingly, combined inhibition of DNA-PK and Pol θ restores radiosensitivity in RPE1 cells, as well as in two p53-mutant breast cancer models.

The role of p53 in modulating DSB repair is complex and incompletely understood. Prior work by Moureau *et al.* suggested that p53-deficient cells favor HR over NHEJ due to impaired recruitment of 53BP1 to DSBs (22). Interestingly, we also observed reduced 53BP1 foci formation within 30 min after NCS treatment in p53-deficient cells (see Figure 2D). However, this difference was largely abolished by low-dose DNA-PKi treatment—indicating that the reduced level of 53BP1 foci formation in p53-deficient cells is most likely due to rapid NHEJ-mediated repair, rather than an impairment in 53BP1 recruitment. Given that NHEJ repair of chromosomal DSBs can occur as early as within 15 min, it seems plausible that accelerated NHEJ-mediated repair of a subset of DSBs may account for the observed reduction in 53BP1 foci formation (52). We did not examine a potential role for HR in suppressing S-phase DSBs, due to a lack of clinical-grade inhibitors that target this pathway. However, the significant impact of DNA-PK and/or Pol θ inhibition on increasing S-phase DSB burden indicates that HR is insufficient for maintaining low levels of S-phase DSBs in p53-deficient cells.

Prior studies have demonstrated a role for p53 in the inhibition of mutagenic NHEJ and MMEJ, the latter of which is now appreciated to be predominantly Pol θ dependent (23,29). Our findings are consistent with these prior reports, and implicate both DNA-PK and Pol θ activity during S phase as critical mediators of radioresistance in p53-deficient cells. The profound effect we observed with partial DNA-PK inhibition (∼50% kinase inhibition using 0.5 μM NU7441) on S-phase DSBs, with a lesser effect on G1-phase DSBs, suggests an S-phase-specific function for DNA-PK in mediating DSB repair. One possibility is that DNA-PK may be particularly important in early S phase, when sister chromatids are not broadly present. Notably, we observed a prominent peak of unrepaired DSBs just as p53-deficient cells transitioned from G1 to S phase (see Figure 2H). DSBs arising in S phase are also subject to end resection that results in a 3′ single-stranded overhang, and short-range resection mediated by Mre11/CtIP can occur within minutes of DSB formation (53). DNA-PK may be necessary—possibly in collaboration with the Artemis nuclease—to cleave these overhangs to blunt ends that are amenable to NHEJ repair (54). DNA-PK has also been implicated in Artemis-independent repair activities that suppress DNA damage and chromosomal instability in S/G2 phases (55). Further mechanistic investigation of how DNA-PK suppresses S-phase DSBs is necessary to clarify the accelerated repair phenotype observed in p53-deficient cells.

Our findings also establish an important role for Pol θ/TMEJ in repairing S-phase DSBs in p53-deficient cells, which is consistent with another recent study using a complementary pro-B-cell model system (56). Pol θ has emerged as the predominant mediator of microhomology-mediated, error-prone, alternative end joining repair in mammalian cells, although its contribution to DSB repair in most ‘normal’ cells is minor (44). Intriguingly, p53 deficiency has recently been shown to stimulate greater recruitment of Pol θ to stalled replication forks, which represents a possible mechanism for Pol θ-mediated repair of S-phase DSBs (57). We observed *POLQ* mRNA overexpression in p53-deficient RPE1 cells, as well as in human cancers with *TP53* gene alterations. The upregulation of *POLQ* mRNA transcription is predominantly indirect, since acute knockdown of *TP53* only resulted in a modest increase in *POLQ* expression. *POLQ* overexpression has also been observed in cancers with *BRCA1*/*BRCA2* mutation, suggesting that it may represent an adaptive response to elevated replication-associated DSB burden, although the precise mechanism of upregulation remains to be elucidated (43,58). In our study, we were able to confirm that *POLQ* in p53-deficient cells mediates TMEJ signature repair at a site-specific chromosomal break. However, improved reagents for Pol θ protein detection are needed to explore whether heterogeneous Pol θ expression at the single-cell level may explain why only a subset of p53-deficient cells are able to survive therapeutic DSBs in the setting of DNA-PK inhibition. Despite these unresolved questions, our study firmly establishes Pol θ/TMEJ, in addition to DNA-PK, as a critical mediator of DSB repair in S phase that promotes resistance to clastogenic therapy in p53-deficient cells (Figure 6F).

Dual tracking of DSBs and cell cycle state transitions revealed new insights into the relationship between therapeutic DSBs, end joining repair pathways and cell cycle checkpoint activation in p53-deficient cells. As expected, p53 deficiency resulted in loss of the well-established p53- and p21/*CDKN2A*-dependent G1/S checkpoint. In contrast, the DNA damage-induced G2/M checkpoint exhibited both p53-dependent and p53-independent characteristics. In p53-proficient cells, 100 ng/ml NCS alone was sufficient to induce prolonged G2 arrest, whereas in p53-deficient cells the same treatment induced only a transient G2 delay. Partial DNA-PK inhibition resulted in further engagement of the p53-independent G2/M checkpoint, although most cells ultimately progressed into mitosis (see Figures 3F and 4B). Indeed, the most common non-viable cell fate after treatment of p53-deficient cells with NCS plus DNA-PKi was mitotic catastrophe.

In contrast, inhibition of Pol θ during NCS treatment in p53-deficient cells was much more effective in engaging a prolonged G2/M checkpoint, which was further augmented by concurrent DNA-PKi treatment. The biological basis for the differential engagement of G2/M checkpoint in the setting of low-dose DNA-PK versus Pol θ inhibition is currently unclear, and warrants further investigation. The overall burden of DSBs in G2 phase may be higher upon Pol θ inhibition, which may result in persistent activation of the cell cycle checkpoint. Alternatively, cells that lack Pol θ may accumulate a particular type of DNA damage, such as resected DSBs, that may more potently activate ATM/Chk2 and/or ATR/Chk1 signaling that is required for G2/M checkpoint activation. Finally, Pol θ activity in cells undergoing G2 arrest may continue to repair DSBs and ultimately diminish the damage signal that is required to maintain the p53-independent G2/M checkpoint. The role of Pol θ/TMEJ in antagonizing prolonged G2 arrest in p53-deficient cells warrants further investigation, as it represents an important pathway for therapeutic resistance.

A limitation of our study is a lack of comparison between the effects of p53 truncation versus missense mutations. Prior work has suggested gain-of-function activity by some p53 missense hotspot mutations, whereas other studies have asserted dominant negative function without gain-of-function activity (59–61). Although the majority of our experiments were performed in models of p53 deficiency, we validated the additive efficacy of DNA-PK and Pol θ inhibition on restoring radiosensitivity in two breast cancer cell line models with *TP53* missense mutations. Nonetheless, future work will be needed to clarify whether cancers with p53 missense mutations have a distinct profile of sensitivity to DNA damage repair inhibitors than cancers with p53 frameshift mutations.

DNA-PK inhibitors are currently undergoing early-stage clinical trial investigation in conjunction with DNA damaging radiotherapy or chemotherapy. Due to dose-limiting toxicities, the clinically achievable doses of DNA-PK inhibitors may only partially inhibit this repair pathway. Our study indicates that while partial DNA-PK inhibition has a modest impact on the viability of p53-proficient cells after radiomimetic treatment, it potently stimulates cell death via mitotic catastrophe in p53-deficient cells, as has recently also been demonstrated in a panel of human cancer cell lines (62). Thus, our study supports the investigation of DNA-PK inhibitors administered in combination with DNA damaging therapy (including radiotherapy) in patients with p53-deficient cancers. However, our findings also indicate that Pol θ/TMEJ activity is a major intrinsic resistance pathway that limits the efficacy of low-dose DNA-PKi therapy as a radiosensitizer. Pol θ has emerged as an attractive therapeutic target due to its hyperactivity in cancers with perturbed DNA damage responses, and its non-essential role in most normal tissue cells (28,43). As clinical-grade inhibitors of Pol θ are currently in development (58), our study suggests that combined inhibition of both DNA-PK and Pol θ represents a promising strategy to reverse therapeutic DNA damage resistance in p53-deficient cancers.

## DATA AVAILABILITY

Further information and requests for resources and reagents should be directed to and will be fulfilled by the lead contact, Gaorav Gupta (gaorav_gupta@med.unc.edu). All unique/stable reagents generated in this study are available from the lead contact with a completed material transfer agreement. All raw live cell imaging files will be deposited in Mendeley Data and data accession number will be available upon acceptance, and they can also be requested from lead contact.

## Supplementary Material

zcaa038_Supplemental_Files
