# Supplementary material for: Dual inhibition of DNA-PK and DNA polymerase theta overcomes radiation resistance induced by p53 deficiency
Source: NAR Cancer. 2020 Dec 21;2(4):zcaa038. doi: 10.1093/narcan/zcaa038 (PMC7751686; doi:10.1093/narcan/zcaa038)
Supplement: zcaa038_Supplemental_Files [file zcaa038_supplemental_files.zip › Supplementary Table 1_vR2.docx]

Supplementary Table S1: Key Resources

| REAGENT or RESOURCE | SOURCE | IDENTIFIER |
| --- | --- | --- |
| **Antibodies** | | |
| F(ab)2-Goat anti-Rabbit IgG (H+L) Cross-Adsorbed Secondary Antibody, Alexa Fluor 633 (1:10,000 for IF) | Thermo Fisher Scientific | Cat# A-21072, RRID:AB_2535733 |
| Chicken anti-Mouse IgG (H+L) Cross-Adsorbed Secondary Antibody, Alexa Fluor 488, (1:10,000 for IF) | Thermo Fisher Scientific | Cat# A-21200, RRID:AB_2535786 |
| Mouse Anti-beta-Actin Monoclonal Antibody, Unconjugated, Clone AC-15 (1:10,000 for WB) | Sigma-Aldrich | Cat# A1978, RRID:AB_476692 |
| Rabbit Anti-53BP1 Polyclonal Antibody (1:500 for IF) | Bethyl | Cat# A300-272A, RRID:AB_185520 |
| Mouse Anti-p53 (1C12) mAb Antibody (1:1000 for WB) | Cell Signaling Technology | Cat# 2524, RRID:AB_331743 |
| Rabbit Anti-phosphorylated Histone H2AX (γ-H2AX) Polyclonal Antibody (1:500 for IF) | Trevigen | Cat# 4418-APC-100 |
| **Chemicals, Peptides, and Recombinant Proteins** | | |
| DNA-PKi | SelleckChemicals | NU7441 (KU-57788) |
| Neocarzinostatin (NCS) 100ng/mL ~ 8.6nM | SigmaAldrich | N9162-100UG |
| Trypsin EDTA | Gibco | 25200-056 |
| Polyethylenimine, Linear (MW 25,000) | Polysciences | 23966 |
| Bovine Serum Albumin | Fisher Scientific | BP9706-160 |
| Corning® Cell-Tak™ and Tissue Adhesive | Corning | 354240 |
| RNAiMax | ThermoFisher | [13778100](https://www.thermofisher.com/order/catalog/product/13778100) |
| **Critical Commercial Assays** | | |
| PlasmoTest | Invitrogen | REP-PT1 |
| RNAeasy Plus Mini Kit | Qiagen | 74136 |
| Comet Assay Kit | Trevigen | 4250-050-K |
| Q5® Hot Start High-Fidelity 2X Master Mix | NEB | M0494S |
| NEBuilder® HiFi DNA Assembly Master Mix | NEB | E2621L |
| TOPO® TA Cloning® Kit for Sequencing | Invitrogen | 450030 |
| T4 DNA Ligase | NEB | M0202S |
| EdU-Click 594 | baseclick | BCK-Edu594 |
| NEON Electroporation Kit | ThermoFisher | MPK1025 |
| Cas9 Protein and TracrRNA for Alt-R Electroporation | IDT | Cas9 (s.p. high fidelity) #1081060  TracrRNA  #1072532 |
| **Experimental Models: Cell Lines** | | |
| hTERT-RPE1-Tricolor Reporter  (PCNA-mCherry, 53BP1-mVenus, H2B-mTurquoise) | Gift from Dr. Jeremy Purvis | (See Citations) |
| hTERT-RPE1 | ATCC | ATCC^®^ CRL-4000^™^ |
| hTERT-RPE1-*TP53-/-* | This paper |  |
| hTERT-RPE1-*TP53-/-POLQ-/-* | This paper |  |
| **Oligonucleotides (sgRNAs and Primers)** | | |
| sgLBR | GCCGATGGTGAAGTGGTAAG | Synthesized at: IDT |
| sgTP53_Exon2 | TCGACGCTAGGATCTGACTG | IDT |
| sgTP53_Dwnstream_Intron | GAAACTGTGAGTGGATCCAT | IDT |
| sgPOLQ_1 | ACTACTCTCAGCTTGA | IDT |
| sgPOLQ_2 | TCAGGAGCATTGCAGCAGAG | IDT |
| LBR_Fwd | AAATGGCTGTCTTTCCCAGTAA | EtonBio |
| LBR_Rev | ACGCAGTGGCTAAATCATCC | EtonBio |
| hPOLQ RTqPCR Primer Fwd | AGGTGGGCTTTCTCCTACTA | EtonBio |
| hPOLQ RTqPCR Primer Rev | CACACTGCTACAGGACGAATAA | EtonBio |
| hβ-actin RTqPCR Primer Fwd | CACCAACTGGGACGACAT | EtonBio |
| hβ-actin RTqPCR Primer Rev | ACAGCCTGGATAGCAACG | EtonBio |
| TP53 RTqPCR Primer Fwd | GAGGTTGGCTCTGACTGTACC | EtonBio |
| TP53 RTqPCR Primer Rev | TCCGTCCCAGTAGATTACCAC | EtonBio |
| CDKN1A RTqPCR Primer Fwd | TCACTGTCTTGTACCCTTGTGCTT | EtonBio |
| CDKN1A RTqPCR Primer Rev | AGAAATCTGTCATGCTGGTCTGCC | EtonBio |
| ONTARGET plus Human TP53 Si-RNA SMARTPOOL | Horizon Discovery (previously Dharmacon) | L-003329-00-0010 |
| ONTARGET plus Human POLQ Si-RNA SMARTPOOL | Horizon Discovery (previously Dharmacon) | L-015180-01-0005 |
| ONTARGET plus NON-TARGETTING control siRNAs SMARTPOOL | Horizon Discovery (previously Dharmacon) | D-001810-10-05 |
| ESR1 Genomic Locus Fwd Primer | ATCTGTACAGCATGAAGTGCAAGA | EtionBio |
| ESR1 Genomic Locus Rev Primer | CTAGTGGGCGCATGTAGGC | EtonBio |
| ESR1 Genomic Locus Probe | T+C+T +AT+G +A+CC TG (Locked nucleic acid probe with HEX) | IDT (LNA : Locked Nucleic Acid Probe) |
| LBR Locus Probe | TGAGATTGAATGTAGCCTTTCTGGCCCTAA (with FAM) |  |
| **LBR Nested Sequencing Primers**  Purple -> Binds genomic DNA  Green -> Phasing portion of primer  chr1:225423928-225424162  Size:   235 base pairs  Forward Primer:  114 base pairs left of cut  Reverse Primer:  123 base pairs right of cut  Rcomp = reverse complimentary    TCAATTCAAGCTCTGTTCCATCTTTATACTTCACAGTGTAAAGCTGGGAGGTGCTGTCGTGGCTCAGAATTTCTACTTCATAATAAAGTGAACTCCCAGGCCATCGA**CCT***CT****TA****CCACTTCACCATCGGC*AAATTTCCTACTTGGCATTTTCTATAATTAACCTGAATAGTTTTAAAGAAAAAAATTTGAGTCAATACATACACATTTATGTATTCGTCTTTTTCCACAGGCTGA       \| **Primer Name** \| **Orientation** \| **Location** \| **Sequence** \| \| --- \| --- \| --- \| --- \| \| LBR2.1 F0 \| Forward \| chr1:225423928-225423949 \| CGACGCTCTTCCGATCTTCAATTCAAGCTCTGTTCCATC \| \| LBR2.1 F1 \| Forward \| chr1:225423927-225423949 \| CGACGCTCTTCCGATCTTTCAATTCAAGCTCTGTTCCATC \| \| LBR2.1 F2 \| Forward \| chr1:225423927-225423949 \| CGACGCTCTTCCGATCTCTTCAATTCAAGCTCTGTTCCATC \| \| LBR2.1 F3 \| Forward \| chr1:225423927-225423949 \| CGACGCTCTTCCGATCTACTTCAATTCAAGCTCTGTTCCATC \| \| LBR2.1 F4 \| Forward \| chr1:225423927-225423949 \| CGACGCTCTTCCGATCTGACTTCAATTCAAGCTCTGTTCCATC \| \| LBR2.1 F5 \| Forward \| chr1:225423927-225423949 \| CGACGCTCTTCCGATCTAGACTTCAATTCAAGCTCTGTTCCATC \| \| LBR2.1 R0 \| Rcomp \| chr1:225424162-225424143 \| CGTGTGCTCTTCCGATCTTCAGCCTGTGGAAAAAGACG \| \| LBR2.1 R1 \| Rcomp \| chr1:225424163-225424143 \| CGTGTGCTCTTCCGATCTATCAGCCTGTGGAAAAAGACG \| \| LBR2.1 R2 \| Rcomp \| chr1:225424164-225424143 \| CGTGTGCTCTTCCGATCTGATCAGCCTGTGGAAAAAGACG \| \| LBR2.1 R3 \| Rcomp \| chr1:225424165-225424143 \| CGTGTGCTCTTCCGATCTTGATCAGCCTGTGGAAAAAGACG \| \| LBR2.1 R4 \| Rcomp \| chr1:225424166-225424143 \| CGTGTGCTCTTCCGATCTCTGATCAGCCTGTGGAAAAAGACG \| \| LBR2.1 R5 \| Rcomp \| chr1:225424167-225424143 \| CGTGTGCTCTTCCGATCTAC \| | | |
| **Software and Algorithms** | | |
| Python ≥v3.5 | *G*. *van Rossum*, *Python tutorial*, *Technical Report CS*-*R9526*, *Centrum voor Wiskunde* en *Informatica* (CWI), *Amsterdam*, May *1995* | https://www.python.org/ |
| Flow Jo | *FlowJo™ Software (Mac) [proliferation assay analysis] Becton, Dickinson and Company; 2019.* | <https://www.flowjo.com/> |
| Graphpad Prism v8 | N.A. | https://www.graphpad.com/ |
| Fiji | *Schindelin, J.; Arganda-Carreras, I. & Frise, E. et al. (2012), “*[*Fiji: an open-source platform for biological-image analysis*](http://www.nature.com/nmeth/journal/v9/n7/full/nmeth.2019.html)*”, Nature methods****9(7)****: 676-682,*[*PMID 22743772*](https://www.ncbi.nlm.nih.gov/pubmed/22743772?dopt=Abstract)*, doi:*[*10.1038/nmeth.2019*](http://dx.doi.org/10.1038%2Fnmeth.2019) ([on Google Scholar](http://scholar.google.com/scholar?cluster=17249863664147333646)). | https://imagej.net/Fiji#Downloads |
| CellProfiler | *CellProfiler Program Citation:*  McQuin C, Goodman A, Chernyshev V, Kamentsky L, Cimini BA, Karhohs KW, Doan M, Ding L, Rafelski SM, Thirstrup D, Wiegraebe W, Singh S, Becker T, Caicedo JC, Carpenter AE (2018). CellProfiler 3.0: Next-generation image processing for biology. PloS Biol. 16(7):e2005970 / [doi](https://doi.org/10.1371/journal.pbio.2005970). PMID: 29969450 (Research article)  *Analyst Software Citation:*  Jones TR, Kang IH, Wheeler DB, Lindquist RA, Papallo A, Sabatini DM, Golland P, Carpenter AE (2008) CellProfiler Analyst: data exploration and analysis software for complex image-based screens. BMC Bioinformatics 9(1):482/doi: 10.1186/1471-2105-9-482. PMID: 19014601 PMCID: PMC2614436 | [www.cellprofiler.org](http://www.cellprofiler.org) |
| NIS Elements AR software |  | <https://www.nikon.com/products/microscope-solutions/lineup/img_soft/nis-elements/> |
| SnapGene software v4.3.4 | GSL Biotech | https://www.snapgene.com |
| Open Comet v1.3.1 | BM Gyori, G Venkatachalam, PS Thiagarajan, D Hsu and MV Clement. “OpenComet: An automated tool for comet assay image analysis”,  Redox Biology, 2:457-465, 2014. | http://www.cometbio.org |
| ScarMapper |  | https://github.com/pkMyt1/ScarMapper.git |
| Additional Image Analysis Scripts | Code EV1  Supplementary and Code Availability (MATLAB scripts) | PMID: 30886052  PMID: 29102360 |
| **Other** | | |
| Genes | www.Ensembl.org | Ensembl v91 |
