# Supplementary material for: Dual inhibition of DNA-PK and DNA polymerase theta overcomes radiation resistance induced by p53 deficiency
Source: NAR Cancer. 2020 Dec 21;2(4):zcaa038. doi: 10.1093/narcan/zcaa038 (PMC7751686; doi:10.1093/narcan/zcaa038)
Supplement: zcaa038_Supplemental_Files [file zcaa038_supplemental_files.zip › SupplementaryData_vR1.pdf]

Supplementary Figure Legends:

Supplementary Fig 1: A, Schematic of CRISPR target locus in human *TP53* gene. Two sgRNAs were designed to target sites in the terminal region of exon 2 (which encodes the p53 transactivating domain) and a site in the downstream intron with a 36 nucleotide (nt) gap. sgRNAs were complexed with Cas9 in the RNP system and electroporated into RPE cells. **B,** Western Blot of 5 selected single-cell clones that were profiled for p53 protein. **C**, Functional assay evaluating p53 dependent p21 transcriptional responses to treatment of 5Gy IR. RNA from cells exposed to IR were harvested 6 hrs post treatment. **D**, Stable representation of labeled and unlabeled p53 wild-type RPE1 cells after exposure to different doses of IR, and measured at the indicated time points by flow cytometry. **E, F**, Clonogenic survival assays performed in RPE cells (WT vs. *p53-/-* cells) exposed to (**E**) IR and (**F**) NCS. **p<0.01, ***p<0.001, ****p<0.0001 by two tailed t-test.

Supplementary Fig 2: A,**B**, Quantification of cell proliferation from live-cell imaging experiments for (**A**) si-control or (**B**) si-TP53 treated RPEs. Cell counts were normalized to cell numbers at start of imaging. Here we show one representative imaging beacon for each treatment condition (untreated, NCS 100 ng/ ml at 18 hours, and NCS 100 ng/ml + 0.5 µM DNA-PKi at 18 hours). **C**,**D**, Clonogenic survival assays of (**C**) WT RPE1 and (**D**) $TP53^{-/-}$ RPE1 cells treated with NCS +/- 0.5 µM DNA-PKi. *p<0.05, **p<0.01, by two-tailed t-test. **E**, Analysis of RPEs with no exposure to NCS (untreated) but received si-control or si-p53 3 days prior to imaging. No significant differences are seen due to si-treatment alone. **F**, Quantification of mitotic outcomes with no NCS exposure over the course of imaging. DNA-PKi treatment alone without NCS showed little to no additional effect on cells (Chi-squared analysis p>0.05 for each condition).

Supplementary Fig 3: **A,** Time stamped image sequence of apoptotic cell (PCNA-mCherry channel shown). Cells that experienced nuclear degradation during cell cycle prior to mitosis were categorized as “apoptotic cells.” In this sequence a cell in G2 experiences cell death at 27 hours post birth, with indication of mitotic attempt, with nuclear envelope collapse or presence of any daughter cells. **B,** Time stamped image sequence of cell that experienced mitotic catastrophe (PCNA channel shown). Cell undergoes nuclear envelope collapse (24:10), and attempts mitosis, in subsequent images fragmentation of nucleus is clearly visible with no viable daughter cells present. Cell non-viability during mitosis was defined as mitotic catastrophe. **C,** Integral DNA damage burden for si-p53 cells treated with NCS (100 ng/ml) and DNA-PKi (0.5 µM) are calculated and stratified by viable (black) versus non-viable (red) cell cycle outcomes. Area under the curve (AUC) analysis was performed by plotting integral 53BP1 foci counts over time for G1 phase (circles), S/G2 phase burden for G1 treated cells (triangles), and S/G2 burden for S phase treated cells (squares). ****p<0.0001 using a two-tailed t-test.

Supplementary Fig 4: **A,** qRT-PCR analysis of *TP53* (left panel) and *POLQ* (right panel) mRNA expression 48 hours after transfection of RPE1 cells with si-Control versus si-TP53. *p<0.05, ****p<0.0001 by two-tailed t-test. **B,** Confirmation of *POLQ* mRNA knockdown 48 hours after transfection of si-POLQ in RPE1 cells. **C**, Inhibition of TMEJ activity 48 hours after si-POLQ transfection, measured using an extrachromosomal TMEJ substrate assay. **p<0.01, ***p<0.001, by two-tailed t-test. **D**, Schematic of CRISPR target locus in human *POLQ* gene. Two sgRNAs were designed to target sites in the polymerase domain, with an 87 nucleotide (nt) gap. sgRNAs were complexed with Cas9 in RNP system and electroporated into $TP53^{-/-}$ RPE cells to generate a double knockout cell line. The lower panels show Sanger sequencing analysis of the CRISPR edited locus in *Polq-/-* RPE clones**.** The locus of interest was PCR-amplified and cloned into a TOPO vector for sequencing analyses. Each line of sequence shown was derived from a different TOPO clone and aligned to show differences. Red boxes indicated sgRNAs used for the CRIPSR.

E, POLQ specific substrates were introduced into the *TP53*$^{-/-}$ vs. *TP53*$^{-/-}$*POLQ*$^{-/-}$ RPE1 cells to assess repair efficiency. Products were amplified and characterized by electrophoresis and end joining efficiency was normalized to *TP53*$^{-/-}$ RPE cells. **F,** Schematic showing evaluation of NGS samples by TIDE analysis for efficiency of cleavage at target site across cell lines. **G,** WT RPE (top panel), *TP53*$^{-/-}$ (middle panel), and *TP53*$^{-/-}$*POLQ*$^{-/-}$ (bottom panel) RPE cell sgLBR cutting efficiency. **H**, (Left panel) Schematic representation of digital PCR based locus detection assay, using primers and probes that flank the sgLBR cleavage site, normalized to a genomic control region on Chromosome 6. (Right panel) Normalized locus detection rate in the different genotypes and treatment conditions. Shown are the mean +/- SEM, *p<0.05, **p<0.05 using a two-tailed t-test.

Supplementary Fig 5: A, Illustrative immunofluorescence images in support of Figure 6C, depicting nuclei (DAPI, blue), 53BP1 foci (green), and EdU incorporation (Red). **B,** Quantification of 53BP1 foci per nucleus for the different genotypes and treatment conditions, using all cells (both EdU positive and EdU negative). The magnitude of differences is comparable to the analysis of S phase cells only, shown in Figure 6. **C,** Confirmation of *POLQ* mRNA knockdown after si-POLQ treatment in (left panel) MDA-MB-231 and (right panel) BT-549 cells.

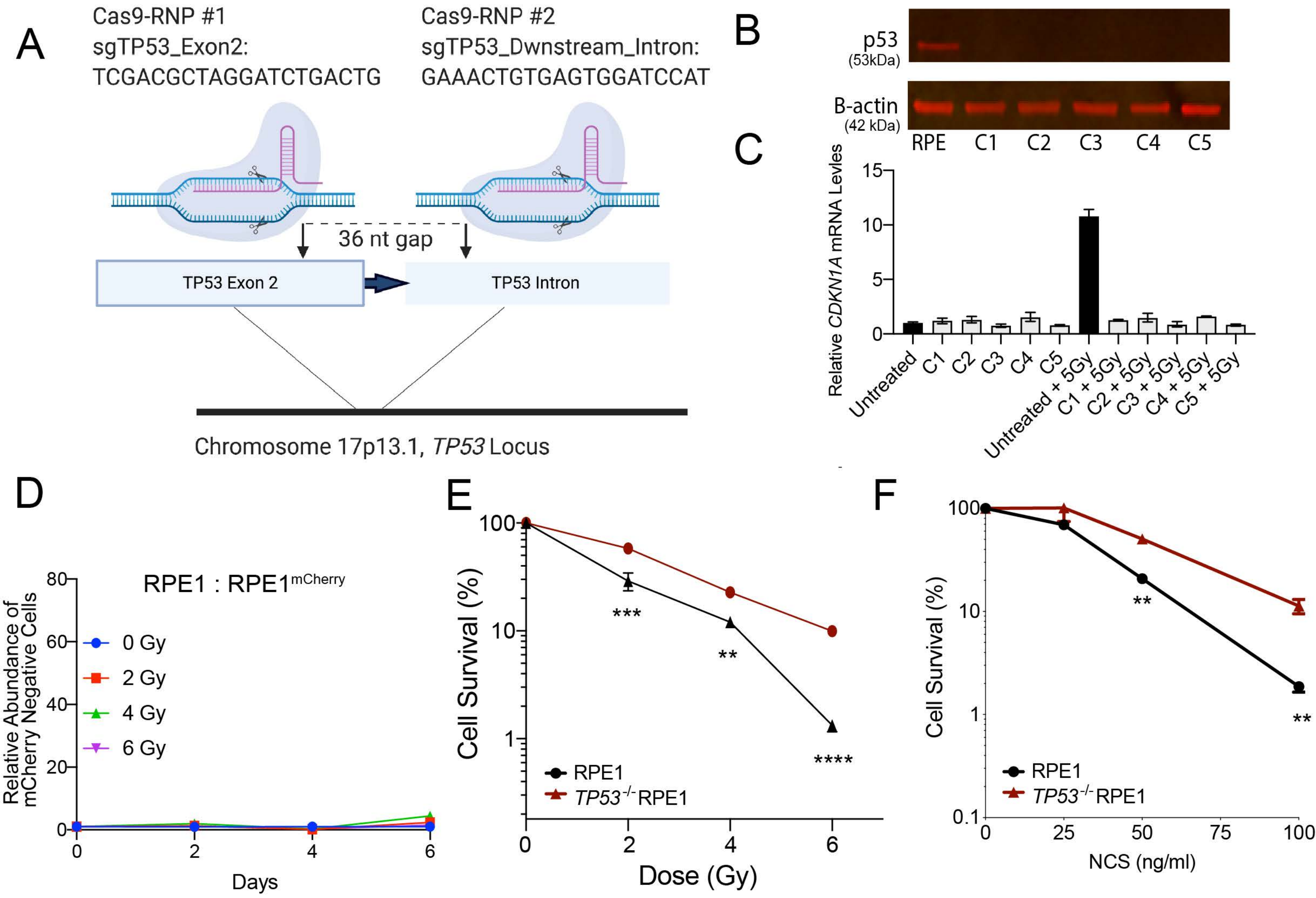

Supplementary Figure 1

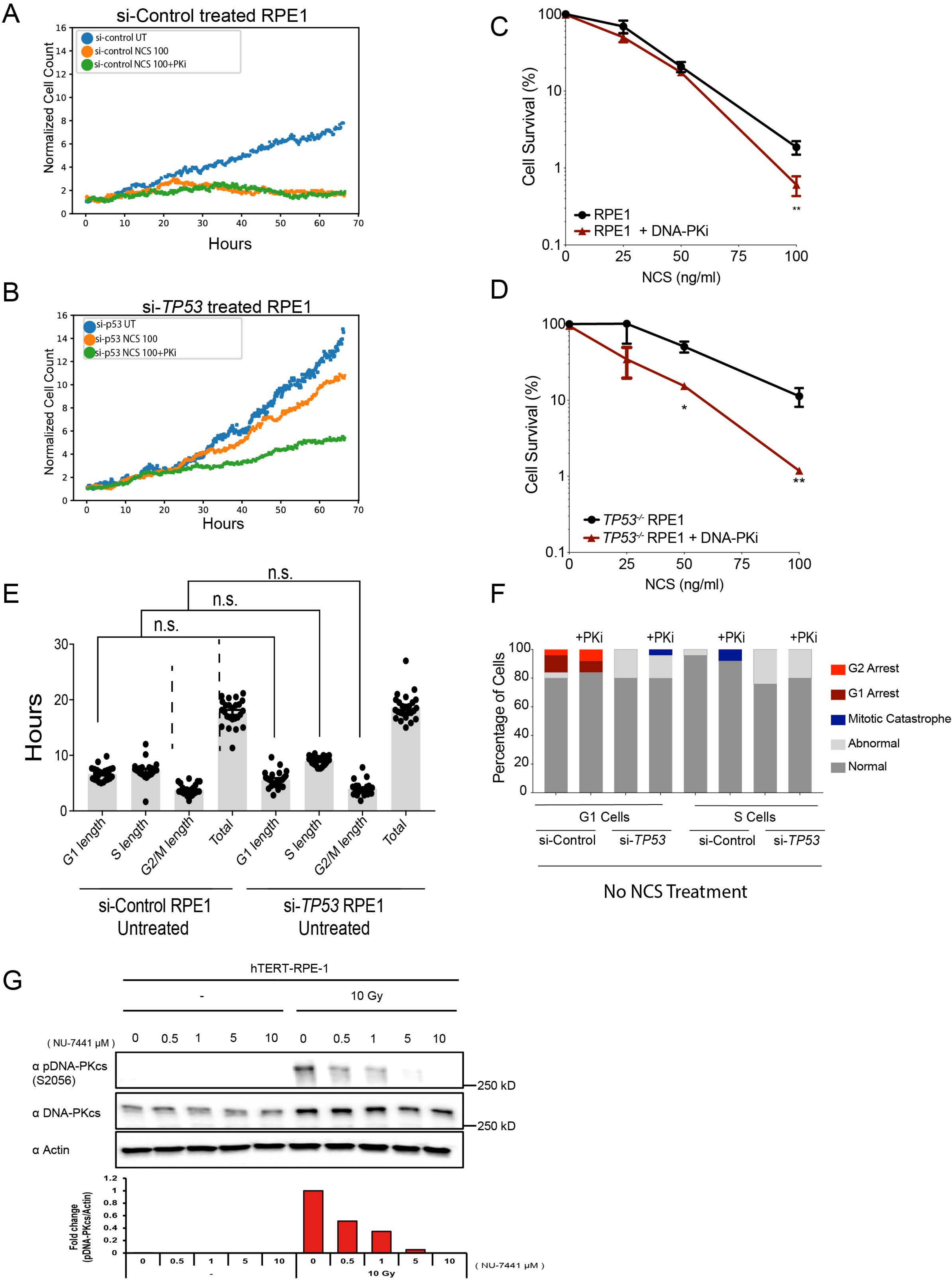

Supplementary Figure 2

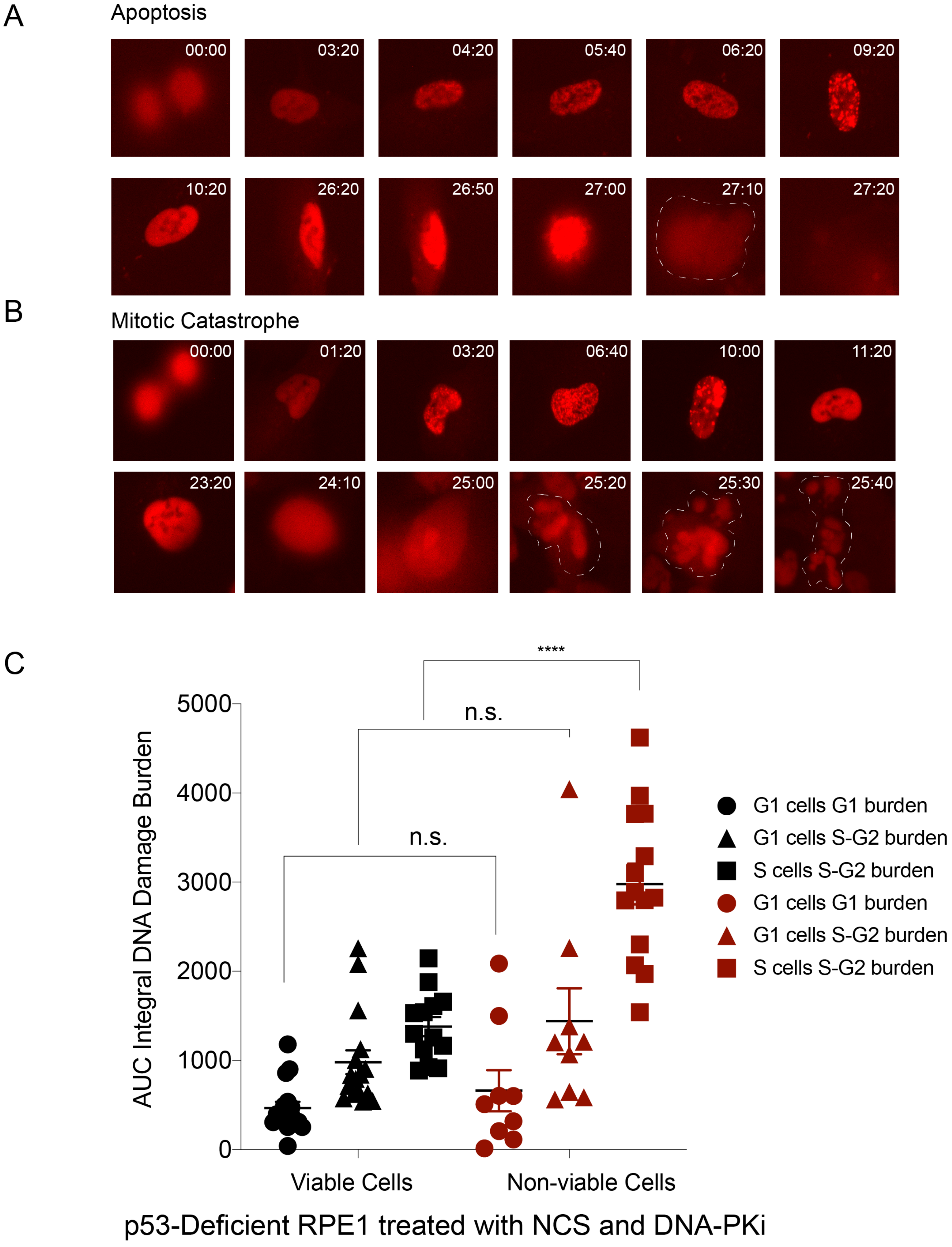

Supplementary Figure 3

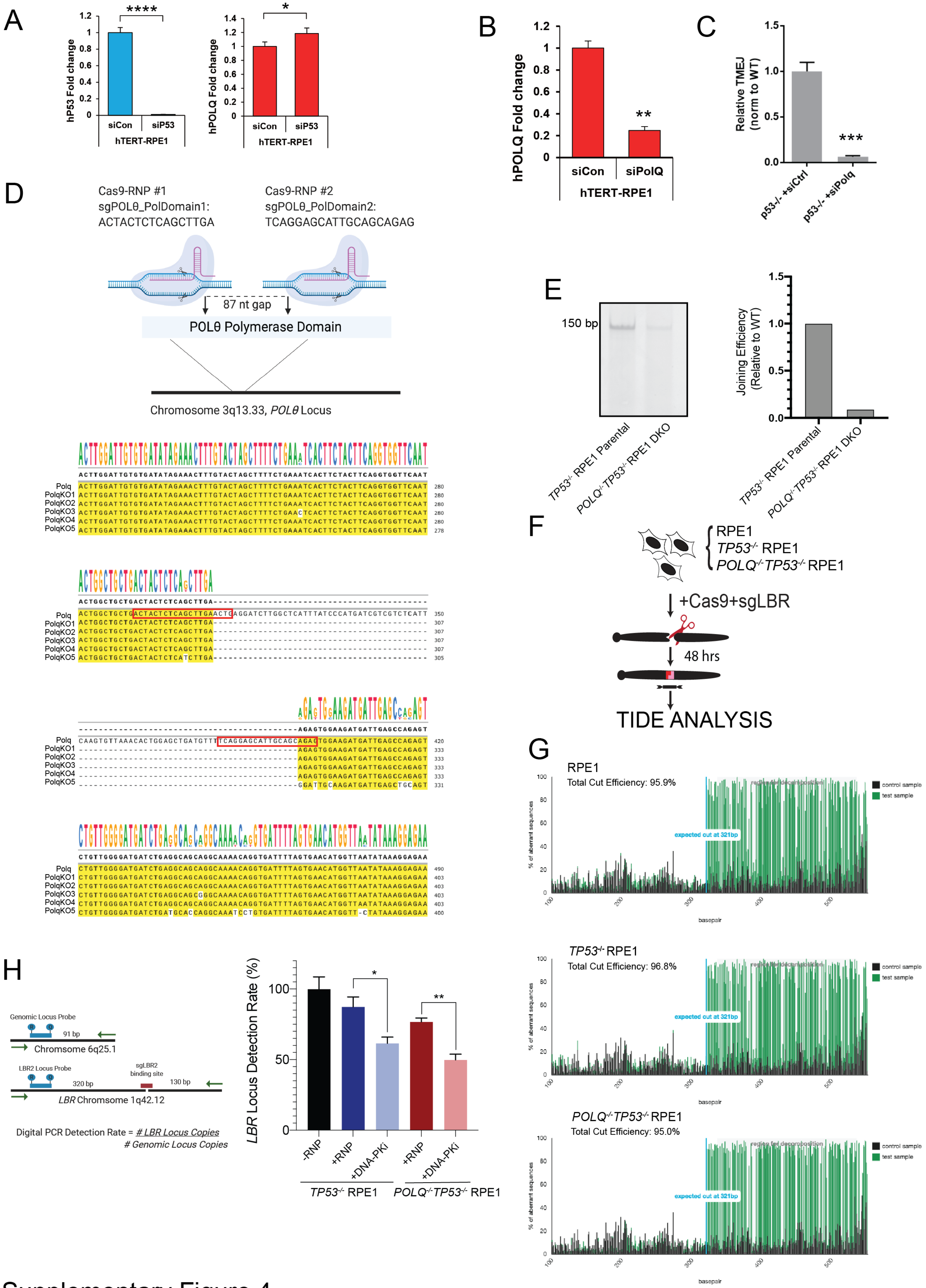

Supplementary Figure 4

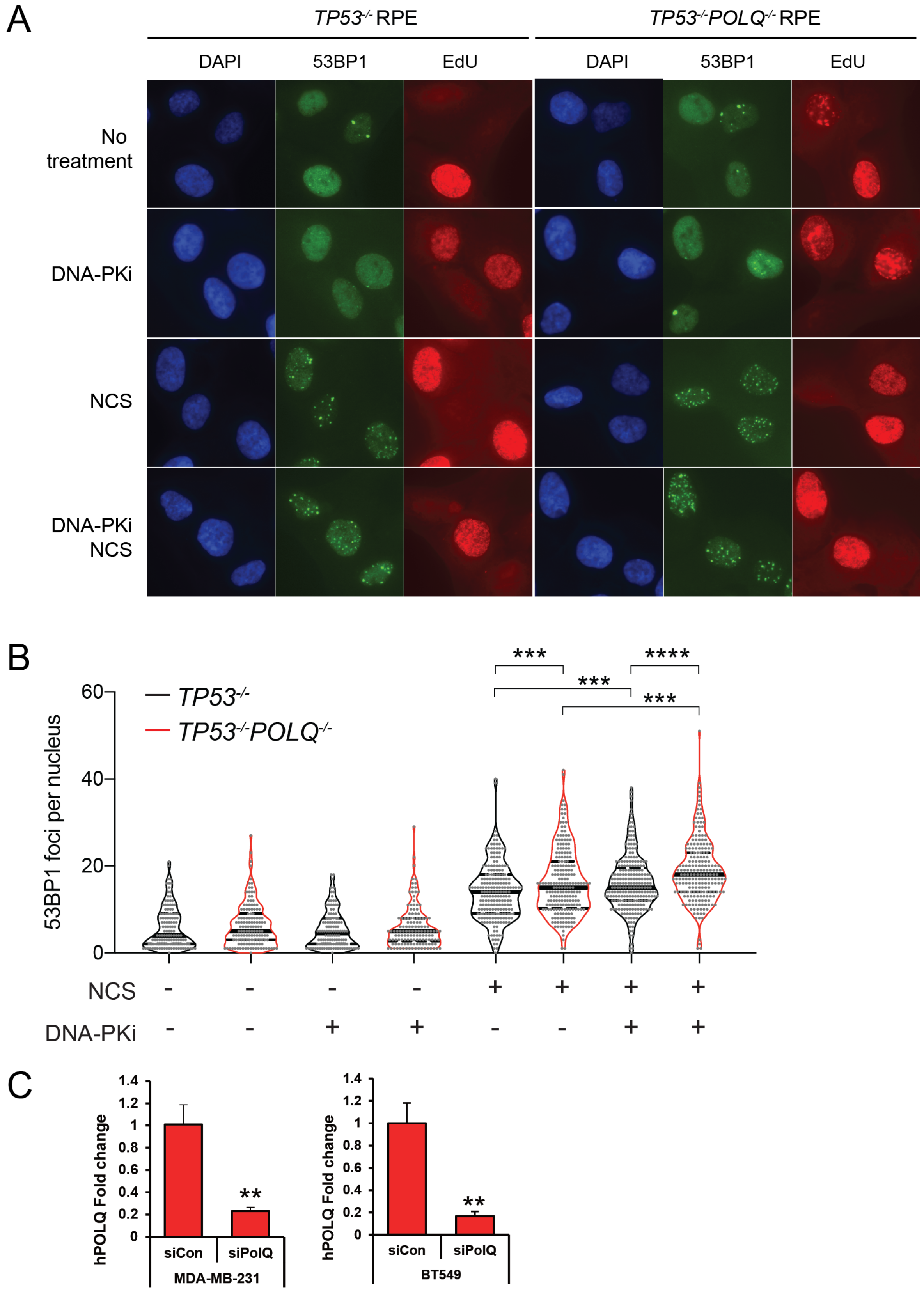

Supplementary Figure 5
